# Making of water soluble curcumin to potentiate conventional antimicrobials by inducing apoptosis-like phenomena among drug-resistant bacteria

**DOI:** 10.1038/s41598-020-70921-2

**Published:** 2020-08-26

**Authors:** Shivangi Yadav, Ashish Kumar Singh, Anand Kumar Agrahari, Kavyanjali Sharma, Anoop Shyam Singh, Munesh Kumar Gupta, Vinod Kumar Tiwari, Pradyot Prakash

**Affiliations:** 1 Bacterial Biofilm and Drug Resistance Research Laboratory, Department of Microbiology, Institute of Medical Sciences, Banaras Hindu University, Varanasi, 221005 India; 2 Department of Chemistry, Institute of Science, Banaras Hindu University, Varanasi, 221005 India; 3 Department of Pathology, Institute of Medical Sciences, Banaras Hindu University, Varanasi, 221005 India

**Keywords:** Drug discovery, Microbiology

## Abstract

The upsurge of multidrug resistant bacterial infections with declining pipeline of newer antibiotics has made it imperative to develop newer molecules or tailor the existing molecules for more effective antimicrobial therapies. Since antiquity, the use of curcumin, in the form of *Curcuma longa* paste, to treat infectious lesions is unperturbed despite its grave limitations like instability and aqueous insolubility. Here, we utilized “click” chemistry to address both the issues along with improvisation of its antibacterial and antibiofilm profile. We show that soluble curcumin disrupts several bacterial cellular processes leading to the Fenton’s chemistry mediated increased production of reactive oxygen species and increased membrane permeability of both Gram-positive and Gram-negative bacteria. We here report that its ability to induce oxidative stress can be harnessed to potentiate activities of ciprofloxacin, meropenem, and vancomycin. In addition, we demonstrated that the soluble curcumin reported herein even sensitizes resistant Gram-negative clinical isolates to the Gram-positive specific antibiotic vancomycin, thereby expanding the antibacterial spectrum of this drug. This work shows that the soluble curcumin can be used to enhance the action of existing antimicrobials against both Gram-positive and Gram-negative bacteria thus strengthening the antibiotic arsenal for fighting resistant bacterial infections for many years to come.

## Introduction

There is a coercing need to find new drug alternatives for the treatment of multidrug resistant (MDR) bacterial infections, which presently affects almost 180 million people across the globe and is anticipated to increase up to 225 million by 2030^1,2^. A number of drugs, which has been introduced in last 2 decades for the treatment of MDR infections helped only for short term in managing infections due to acquisition and dissemination of the newer resistance^3^. As per the recent data mining, the current assessment of the pipeline shows about 42 new antibiotics in development among which 11 are in Phase I clinical trials, 13 in Phase II, 13 in Phase III, four have submitted marketing authorization applications, and only one drug has received a complete response letter. However, given the irrevocability that some of these “in development” antibiotics will be declined for approval, and that resistance will eventually develop with time to those that will be consented for use, it is unblemished that we will have too few drugs to meet current and anticipated patient needs^4^. The global menace of resistance can be understood with the fact that we are helpless against carbapenem-resistant/extended spectrum β-lactamase (ESBL)-producing *Enterobacteriaceae*, *Acinetobacter baumannii*, *Pseudomonas aeruginosa*, and Methicillin resistant *Staphylococcus aureus* as they are resistant to all or nearly all of the antibiotics available today whom the World Health Organization considers critical threats^5^. The studies indicate that the upsurge of resistant strains can only be successfully tackled after targeting the multiple bacterial components and simultaneously repurposing the existing treatment arsenal. Curcumin fitted well in our requirement window^6^.

Curcumin, a polyphenol, is the active ingredient of turmeric, is documented frequently to be highly effective against a wide array of microbial pathogens ranging from *Helicobacter pylori,* and *Escherichia coli* to *Candida albicans* and therefore, indicating its structural tuneability that can be harnessed to concurrently target the multifaceted features of multi-drug resistance (MDR)^7–14^. Although it has activities against most of the pathogenic isolates exhibiting MDR but at significantly higher doses^7,13,15^.

Curcumin has two major salient structural features: presence of phenolic rings and the resonating keto or aldehyde unit^16^. Phenolic rings may disrupt aromatic π−π stacking by interacting with the aromatic residues of the structural proteins while the hydroxyl groups may foster β-sheet breakings and other sugar based interactions via competitive hydrogen bonding^16^. However, one of the foremost limitations of using curcumin as a drug is its poor aqueous solubility, which eventually is responsible for its instability and poor bioavailability^17^. Nevertheless, attempts have been made to increase its aqueous solubility and bioavailability by the nanoparticle-based approach producing micellar and lipid-drug hybrid nanoparticle formulations with improved water/plasma solubility^18^. Nonetheless, the innate toxicity issues cannot be overlooked^19^. The noncovalent formulation approach experiences other issues like limited transport of the micelles, slow drug release, and even leaching which jeopardize its use as frontline therapeutic agent^20–23^. Even the quantum dots of curcumin are also reported which have shown improved stability, solubility, and therapeutic potential^6,19,24^. Apart from this, the attempt of covalent modification of one of the ketone groups in the β-diketone bridge of curcumin with phenyl hydrazine has also been made which resulted in a derivative of curcumin, CNB001. Unfortunately, this derivative was more hydrophobic than the native curcumin^25^.

The facts clearly reveal that the essential issue associated with curcumin is its poor aqueous solubility that has not yet been addressed entirely and hence, there is an undeniable need for better curcumin based unique anti-bacterial/ anti-biofilm agents acting on bacterial membrane/biofilm protein frameworks. We herein report the “Click-based” synthesis of bifunctional curcumin-galactose conjugate with detailed evaluation of its role as direct antimicrobial, antibiofilm as well as its possible use as antibiotic adjuvant. In addition, we further delineated the mechanistic underpinnings of its mode of actions with subsequent detailed biocompatibility evaluation on cell lines as well as on Charles foster rats.

## Results

### Synthesis of water-soluble curcumin

**Figure 1:**
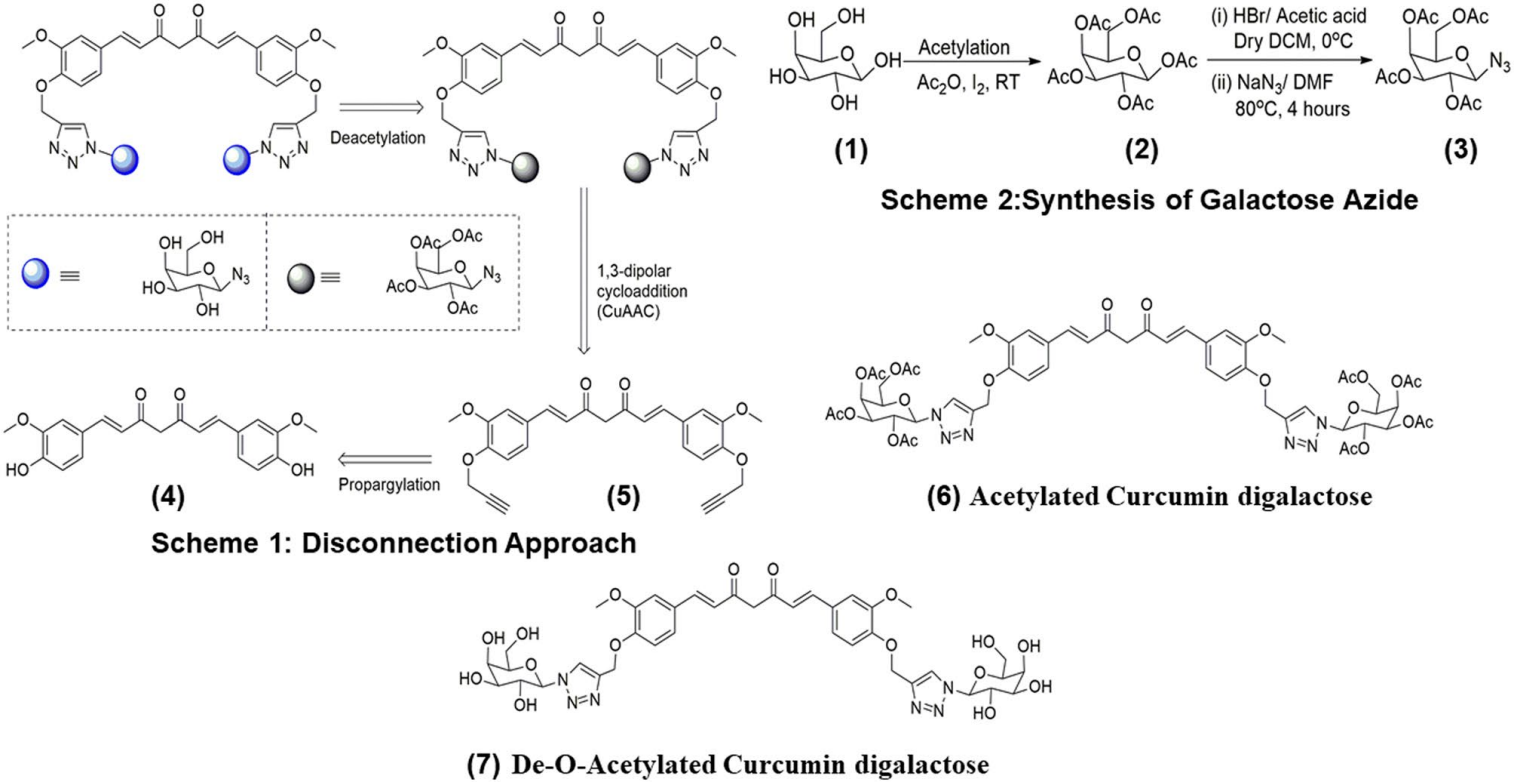
Scheme 1 depicts the disconnection approach for the synthesis of di-galactosylated Curcumin. Scheme 2 depicts the synthesis of galactose azide. (1) galactose, (2) acetylated galactose, (3) galactose azide, (4) curcumin, (5) curcumin di-alkyne, (6) curcumin clicked di acetylated galactose, and (7) de-*O*-acetylated curcumin digalactose.

For the preparation of click inspired galactosylated curcumin derivative 7, we sketch the three-step disconnection approach in Fig. 1. We first of all propargylated the curcumin to form propargylated curcumin, this curcumin derivative underwent 1,3-dipolar cycloaddition reaction with acetylated sugar azide to form glycoconjugate curcumin derivative **6** which made it water soluble when we convert acetyl group to hydroxyl group by deacetylation **7** (for peaks and the related information, see the “Methods” section).

### Solubility comparison between curcumin and galactose conjugated curcumin

The dramatically augmented aqueous solubility of the galactose clicked curcumin was substantiated utilizing UV–Vis spectrometry. Briefly, 5 mg of sCur was vortex-mixed in 1 ml of MilliQ water in micro centrifuge tube to prepare the stock solution. For comparison, native curcumin (5 mg) was also vortex-mixed in 1 ml of MilliQ water in micro centrifuge tube. Both the samples were then centrifuged at 13,000 RPM for 5 min to remove undissolved part if any. Then, 100 µl of sCur was added to 900 µl of MilliQ water and mixed properly. The absorbance profile of the diluted sCur and native curcumin was documented. The molar extinction coefficient was found to be 6.1625 × 10^3^ L mol^−1^ cm^−1^ at λ_max_ 320 nm, whereas for native curcumin, at this wavelength, molar extinction coefficient remained undetermined. Therefore, after comparing the two data sets, almost 11,000 times better aqueous solubility was noted for sCur.

### Determination of minimum inhibitory and minimum bactericidal concentration (MIC and MBC)

Minimum inhibitory concentration of sCur along with test drugs ciprofloxacin, vancomycin, and meropenem against aforementioned bacterial controls and clinical isolates (*Staphylococcus aureus* (ATCC29213), *Staphylococcus epidermidis* (ATCC35984), *Klebsiella pneumoniae* (ATCC700603), *Escherichia coli* (ATCC25922), and *Pseudomonas aeruginosa* (ATCC25619), *Klebsiella pneumoniae* (Lab code: 10,894/2019), Methicillin-sensitive *Staphylococcus aureus* (Lab code: 2,862/2019, MSSA), Methicillin-resistant *Staphylococcus aureus* (MRSA, lab code: 2,859/2019), *Escherichia coli* (Lab code: 507/2019), and *Pseudomonas aeruginosa* (Lab code: 2,412/2019) was determined by broth micro-dilution assay. The MIC of sCur against Gram-positive and Gram-negative MDR isolates was 32 and 64 μg/ml whereas MIC of meropenem, ciprofloxacin, and vancomycin were found to be 128, 64, and 512 μg/ml respectively (Table 1).

**Table 1:** Minimum inhibitory concentration of sCur vis a vis native curcumin/vancomycin/meropenem/ciprofloxacin.

Isolates	Curcumin		Soluble curcumin		Vancomycin		Meropenem		Ciprofloxacin	
	MIC (µg/ml)	MBC (µg/ml)	MIC (µg/ml)	MBC (µg/ml)	MIC (µg/ml)	MBC (µg/ml)	MIC (µg/ml)	MBC (µg/ml)	MIC (µg/ml)	MBC (µg/ml)
*Staphylococcus aureus* (ATCC29213)	128	≥ 1,024	16	64	0.5	2	-	-	0.25	0.5
*Staphylococcus aureus* (MRSA, 2,862/2019)	256	≥ 1,024	32	128	1	2	-	-	32	64
*Staphylococcus epidermidis* (ATCC35984)	128	≥ 1,024	16	32	0.25	1	-	-	< 0.25	0.5
*Klebsiella pneumoniae* (ATCC700603)	256	≥ 1,024	32	128	256	≥ 1,024	0.5	1	0.5	1
*Klebsiella pneumoniae* (10,825/2019)	512	≥ 1,024	32	256	512	≥ 1,024	128	256	64	128
*Escherichia coli* (ATCC25922)	256	≥ 1,024	32	128	256	≥ 1,024	0.25	1	0.25	0.5
*Escherichia coli* (507/2019)	512	≥ 1,024	32	128	512	≥ 1,024	64	128	32	64
*Pseudomonas aeruginosa* (ATCC25619)	512	≥ 1,024	32	256	1,024	≥ 1,024	0.5	1	0.5	1
*Pseudomonas aeruginosa* (2,412/2019)	≥ 1,024	≥ 1,024	64	256	1,024	≥ 1,024	128	256	64	256

### Tissue culture plate based anti-biofilm assay

**Figure 2:**
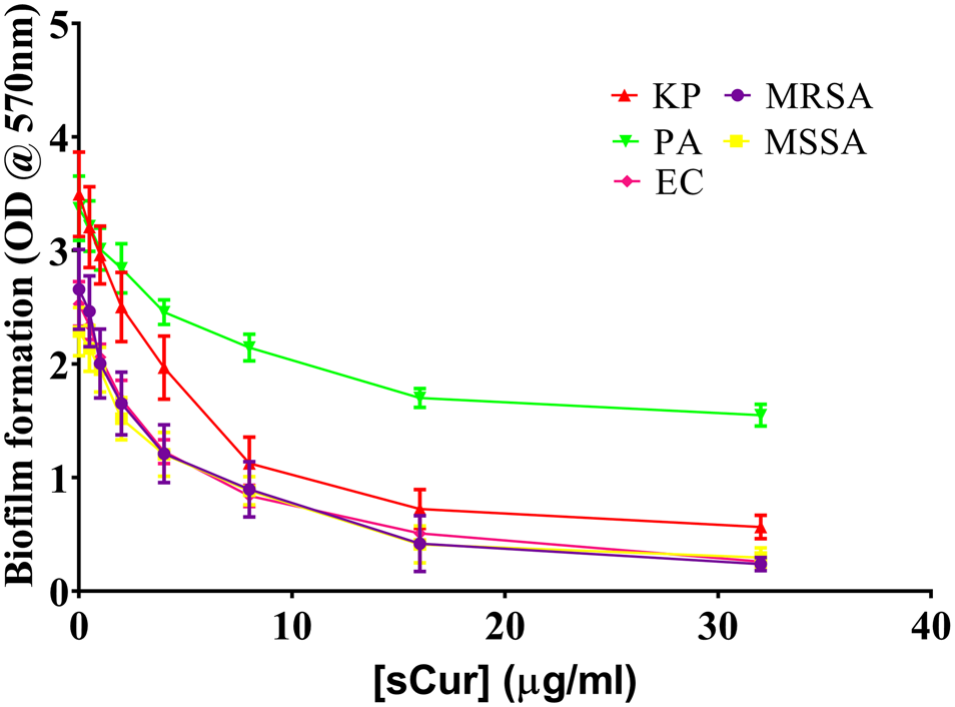
Biofilm reduction curve of soluble curcumin against the multi drug resistant isolates depicting the concentration dependent reduction of biofilm biomass [Graph was generated and analyzed using GraphPad 8.0 (San Diego, CA)].

The sCur mediated biofilm inhibition was dose-dependent against all the tested bacterial isolates. At the concentration 8 µg/ml, 67% biofilm inhibition was realized in case of MRSA clinical isolate (2,859/2019) but, no sooner, the concentration was escalated to 32 µg/ml then, 91% inhibition was observed. While at the same concentration 89%, 87%, and 90% inhibition was noted in *Klebsiella pneumoniae* (1,337/2019), MSSA (2,862/2019) and *E. coli* (507/2019) respectively (Fig. 2). Interestingly, sCur was found comparatively less effective against the biofilms of *Pseudomonas aeruginosa* (2,412/2019) at the said concentration where merely 54% inhibition was realized.

### Combination of sCur with ciprofloxacin, meropenem, and vancomycin exhibited selective synergistic toxicity against bacteria

**Figure 3:**
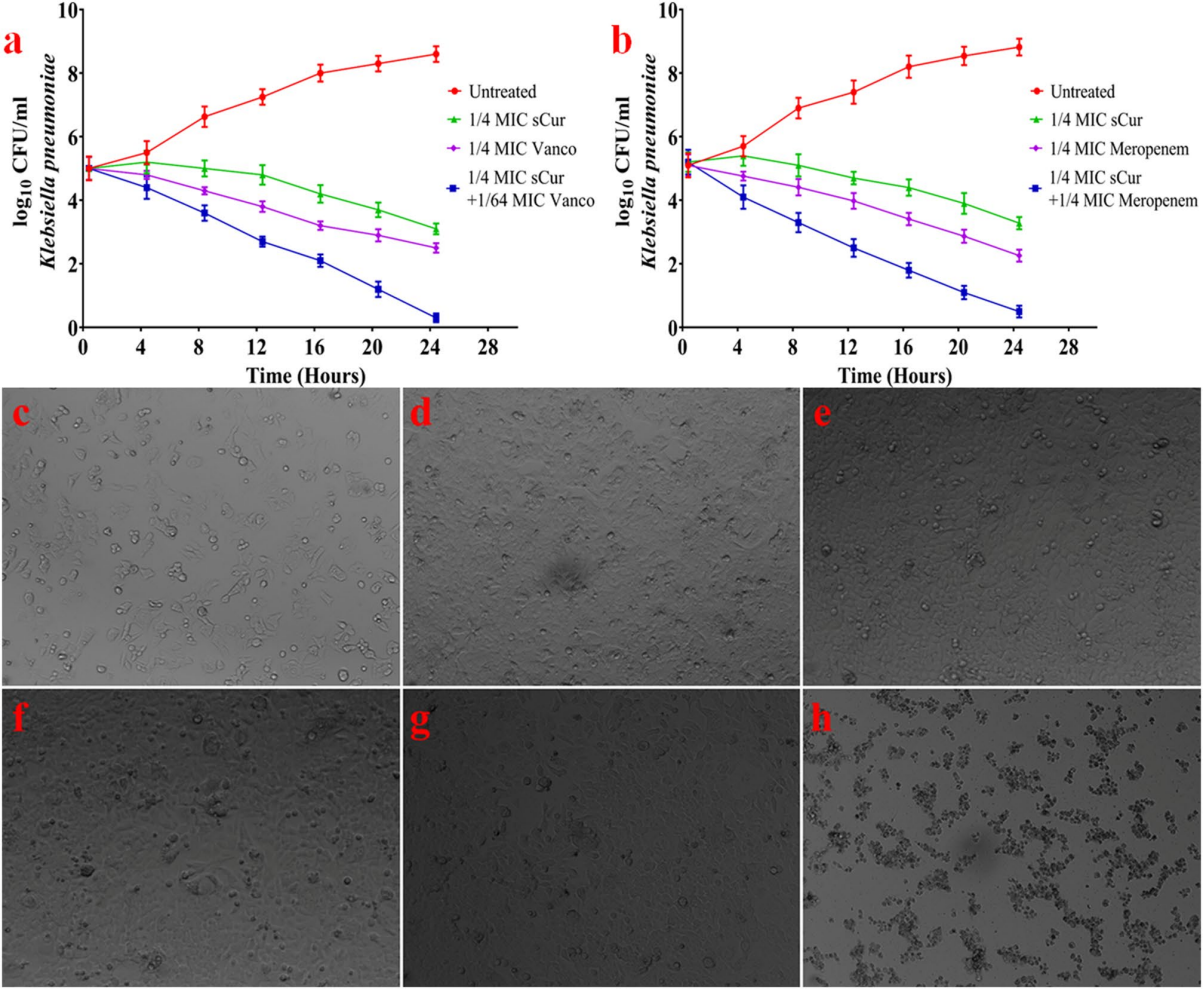
(**a**) Kill curves for log-phase growing multi-drug resistant *Klebsiella pneumoniae* treated with various concentrations of soluble curcumin, vancomycin, and their combination (**b**) Kill curves for log-phase growing multi-drug resistant *Klebsiella pneumoniae* treated with various concentrations of soluble curcumin, meropenem and their combination. (**c**–**h**) Evaluation of cytotoxicity over HCT116 cells post-treatment. (**c**) Untreated HCT116 cells at the time of seeding (**d**) Untreated HCT 116 cells after 48 h (**e**) HCT116 cells treated with 64 µg/ml ciprofloxacin and 8 µg/ml sCur (**f**) HCT116 cells treated with 128 µg/ml meropenem and 8 µg/ml sCur (**g**) HCT116 cells treated with 1,024 µg/ml vancomycin and 8 µg/ml (**h**) HCT116 cells treated with 100 µg/ml Imatinib [Graph was generated and analyzed using GraphPad 8.0 (San Diego, CA)].

The effect of sCur with ciprofloxacin, meropenem, and vancomycin in combination on the growth of aforementioned resistant bacterial isolates was investigated in the microtiter plates by the log_10_ CFU/ml reduction and Bliss model of synergism. Ciprofloxacin alone inhibited drug-resistant *E. coli*, *K. pneumoniae,* and *P. aeruginosa* growth with a minimal inhibition concentration (MIC) of 32, 64, and 64 µg/ml after 18 h treatment, while the addition of 8 µg/ml sCur dramatically decreased the MIC of ciprofloxacin to 1, 2 and 2 µg/ml respectively. Similarly, MIC of ciprofloxacin against MRSA was found to be 32 µg/ml, which reduced to 1 µg/ml after administration of 8 µg/ml sCur. However, the MIC of vancomycin against *E. coli*, *K. pneumoniae,* and *P. aeruginosa* were found to be 512, 512, and 1,024 µg/ml, which dramatically reduced to 8, 16, and 16 µg/ml upon exposure of 8 µg/ml sCur. Similarly, the MIC of meropenem alone was found to be 64, 128, and 128 µg/ml against resistant isolates of *E. coli*, *K. pneumoniae, and P. aeruginosa* respectively, which reduced to 1, 2 and 4 µg/ml post sCur supplementation (Table 2, Fig. 3a, b). However, native curcumin in synergy remained ineffective in the tested concentration range (Supplementary information 3, Table T7).

**Table 2:** Modified minimum inhibitory concentration of vancomycin, meropenem, and ciprofloxacin upon administration with sCur (8 µg/ml) against MDR pathogens.

Isolates	Vancomycin modified MIC (µg/ml)	Meropenem modified MIC (µg/ml)	Ciprofloxacin modified MIC (µg/ml)
*Staphylococcus aureus* (MRSA, 2,862/2019)	0.25	–	1
*Klebsiella pneumoniae* (10,825/2019)	16	2	2
*Escherichia coli* (507/2019)	8	1	1
*Pseudomonas aeruginosa* (2,412/2019)	16	4	2

Meanwhile, 64 µg/ml ciprofloxacin and 8 µg/ml sCur, 128 µg/ml meropenem and 8 µg/ml sCur and 1,024 µg/ml vancomycin and 8 µg/ml sCur in combination showed no synergistic toxicity on human HCT 116 cells (Fig. 3c–g).

**Figure 4:**
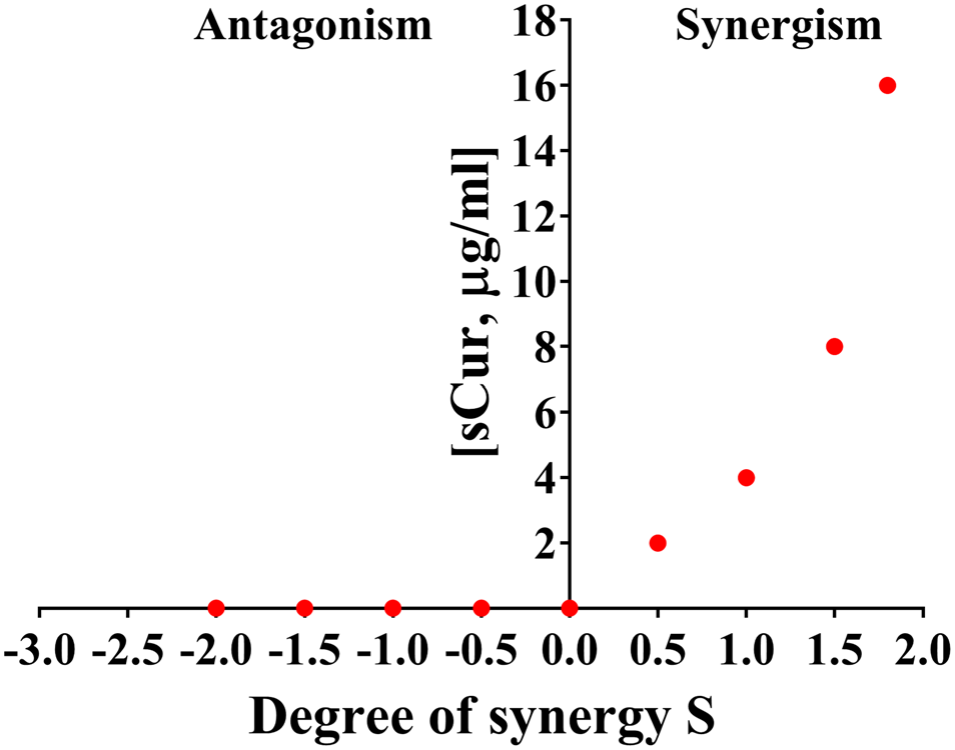
The Bliss Model for Synergy confirms a synergistic effect, between sCur and vancomycin, against *Klebsiella pneumoniae*, Gram-negative bacteria. Degree of synergy quantified, using the Bliss Model for Synergy, after 24 h of treatment with 16 µg/ml vancomycin in combination with sCur at 2, 4, 8, and 16 µg/ml.

This indicates that these combinations exhibit significant selective synergistic toxicity on bacteria over mammalian cells, and the dramatic decrease in MIC of vancomycin, ciprofloxacin, and meropenem against bacteria in the presence of sCur signals the possible feasibility of systemic medical use of sCur (Fig. 4).

### Confocal laser scanning microscopic evaluation of synergistic antibacterial potential of sCur

**Figure 5:**
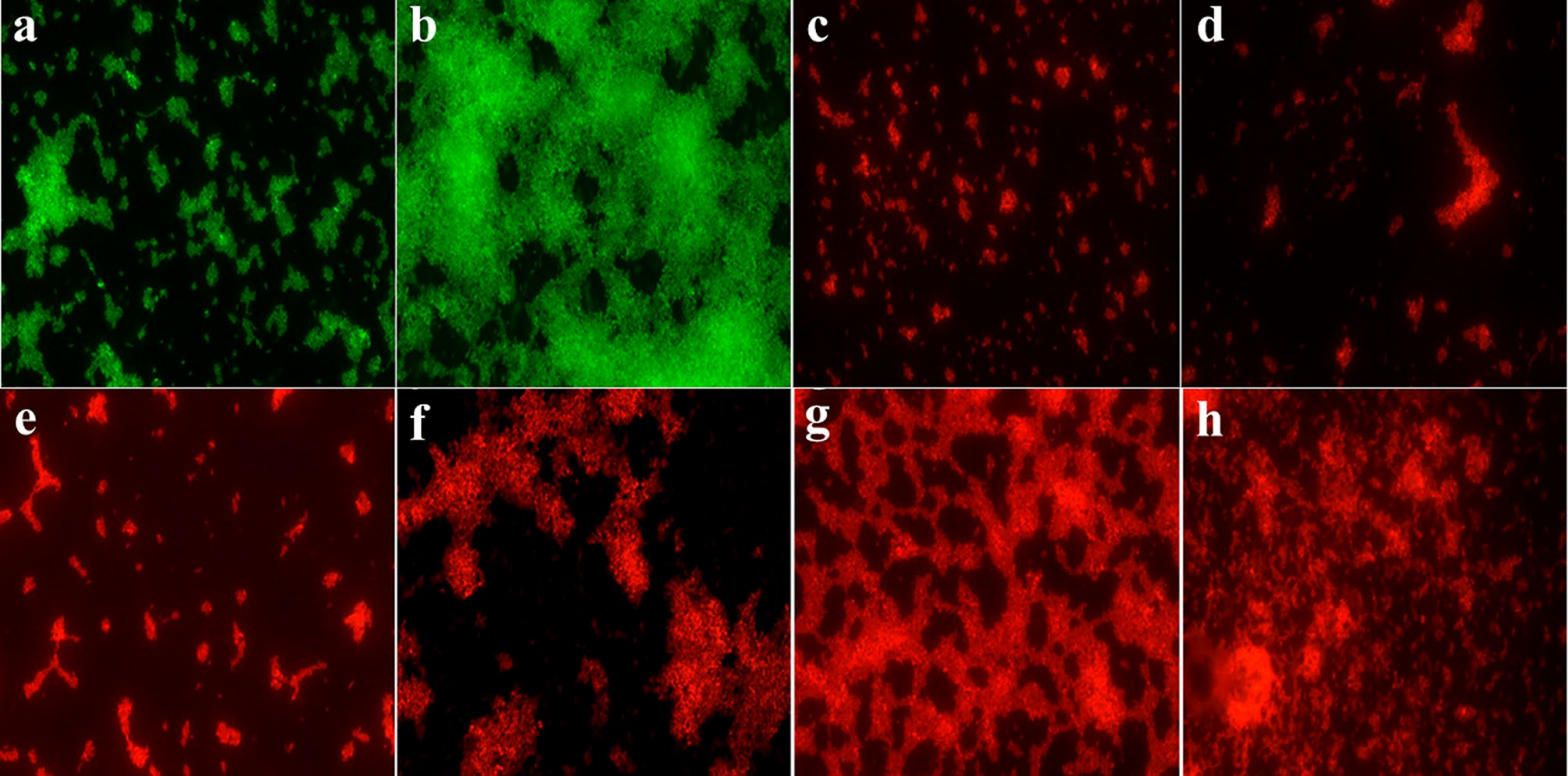
Confocal Microscopic validation of synergism between soluble curcumin and vancomycin over multidrug resistant clinical *Klebsiella pneumoniae.* (**a**) Untreated *Klebsiella pneumoniae* at t = 0 h. (**b**) Untreated *Klebsiella pneumoniae* at t = 18 h. (**c**) Soluble curcumin (8 µg/ml) treated *Klebsiella pneumoniae* at t = 18 h. (**d**) Vancomycin treated (512 µg/ml) treated *Klebsiella pneumoniae* at t = 18 h. (**e**) Soluble curcumin (8 µg/ml) and vancomycin (2 µg/ml) treated *Klebsiella pneumoniae* at t = 18 h. (**f**) Soluble curcumin (8 µg/ml) and vancomycin (4 µg/ml) treated *Klebsiella pneumoniae* at t = 18 h. (**g**) Soluble curcumin (8 µg/ml) and vancomycin (8 µg/ml) treated *Klebsiella pneumoniae* at t = 18 h. (**h**) Soluble curcumin (8 µg/ml) and vancomycin (16 µg/ml) treated *Klebsiella pneumoniae* at t = 18 h.

The synergistic antibacterial effect of sCur and vancomycin on multidrug resistant clinical *Klebsiella pneumoniae* isolate (10,825/2019) was investigated by confocal microscopy. The freshly grown bacteria was then challenged with the sub lethal concentration of sCur (8 µg/ml) and vancomycin (2, 4, 8, and 16 µg/ml) followed by staining with the red fluorescent dye propidium iodide and imaging. Panel a depicts the untreated *Klebsiella pneumoniae* at the start time while panel b is untreated *Klebsiella pneumoniae* after 18 h of growth as stained by syto9, the green dye staining the live cells only (Fig. 5a, b). Interestingly, panel c represents the soluble curcumin (8 µg/ml) treated *Klebsiella pneumoniae* after 18 h (Fig. 5c). Interestingly, the test *Klebsiella* isolate exhibited an intense PI staining the mere concentration of sCur (8 µg/ml) and vancomycin (2 µg/ml), indicating the potential death of the bacterial cells after exposure to the combination of the drugs (Fig. 5f). However, when the concentration of vancomycin was escalated to 4 µg/ml, substantial killing can be perceived with intense red signals (Fig. 5g). The results show an obvious dose-dependent bactericidal activity of the combination evident by the observed increase in the red fluorescent signals in panels e–h (Fig. 5). However, it was noted that, after administering 8 µg/ml soluble curcumin and 8 µg/ml vancomycin (panel h), intensity of red fluorescence further increased, indicating pronounced killing activity. However, the comparative evaluation of native curcumin and vancomycin combination on *Klebsiella pneumoniae* viability at different concentrations reveal no significant effect (Supplementary information 4).

**Figure 6:**
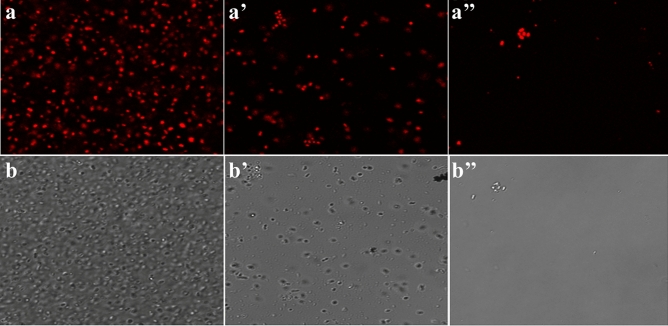
The Confocal Microscopic validation of time-dependent effect of the soluble curcumin (8 µg/ml) and vancomycin (8 µg/ml) combination over multidrug resistant clinical *Klebsiella pneumoniae.* The panels (**a**), (**a’**), and (**a’’’**) represent the confocal micrographs of the isolates incubated with the said combination for 6, 12 and 24 h respectively while the panels (**b**), (**b’**), and (**b’’**) depict the differential interference contrast (DIC) micrographs of the same.

To check the time-dependent effect of the drug combination, we used the sCur (8 µg/ml) and vancomycin (8 µg/ml) combination. By moving down the lane from a to a’’, we may observe the progressive loss of bacterial population indicating the bacterial lysis due to prolonged exposure to the drug combination (Fig. 6a, a’, a’’). This was further validated by differential interference contrast imaging (Fig. 6b, b’, b’’). One can note the rarefaction of bacterial population with the increase in incubation time, almost complete lysis after 24 h (Panel b’’, Fig. 6).

**Figure 7:**
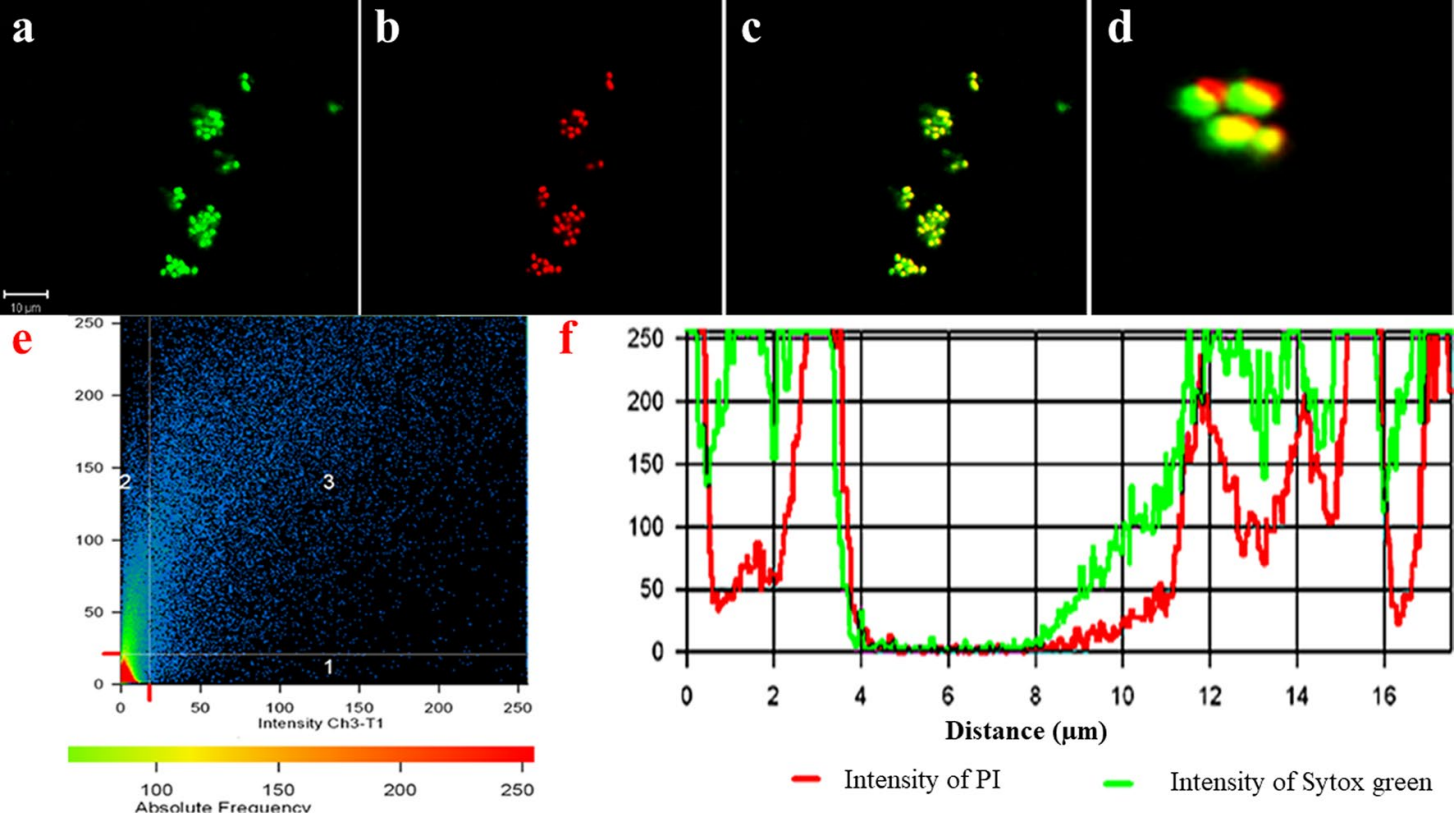
The Confocal Microscopic validation of the soluble curcumin-vancomycin combination mediated bacterial cell membrane using Sytox green-propidium iodide dual staining. (**a**) Post treatment micrograph stained with Sytox green. (**b**) Post treatment micrograph stained with Propidium iodide. (**c**) Merged overlay of panels (**a**) and (**b**). (**d**) Zoomed in bacterial clusters from panel (**c**) to show the precision of overlap of Sytox green and propidium iodide. (**e**) Co-localization map of PI with sytox green. (**f**) Comparative relative intensity and distance plot of sytox green and PI for co-localization validation (all confocal images were analyzed and processed using ZenBlue imaging software).

We realized PI to be localized all around the bacteria indicating the compromised cell membrane. Even the DIC micrographs signaled the same. To further validate our finding we utilized sytox green-PI dual staining to establish the sCur mediated plasma membrane disintegration. Surprisingly, we observed an obvious co-localization of PI with sytox green in bacteria (Fig. 7a–c). This co-localization was signaled by the presence of an intermediary yellow colour (panel d, Fig. 7). In order to check the sharing of co-localization of PI with sytox green, we analyzed the relative intensity and distance plot of the defined region highlighted by red (panel e, f; Fig. 7). The relative intensity of red and green signals retraces their paths in the defined distance frame as evident from the panel f of Fig. 7. This synchronized localization of PI and sytox green distinctly depicts the membrane permeabilization orchestrated by sCur, which ultimately leads to the ooze out of cytosolic content from the bacteria as revealed by the red signals of PI coming out of the cell (panel f, Fig. 7).

The synthesized sCur seems to foster the rupture of the plasma membrane, which ultimately leads to the ooze out of cytosolic content from the bacteria as revealed by the red signals of PI coming out of the cell.

## Mechanistic

### Changes in membrane dynamics

**Figure 8:**
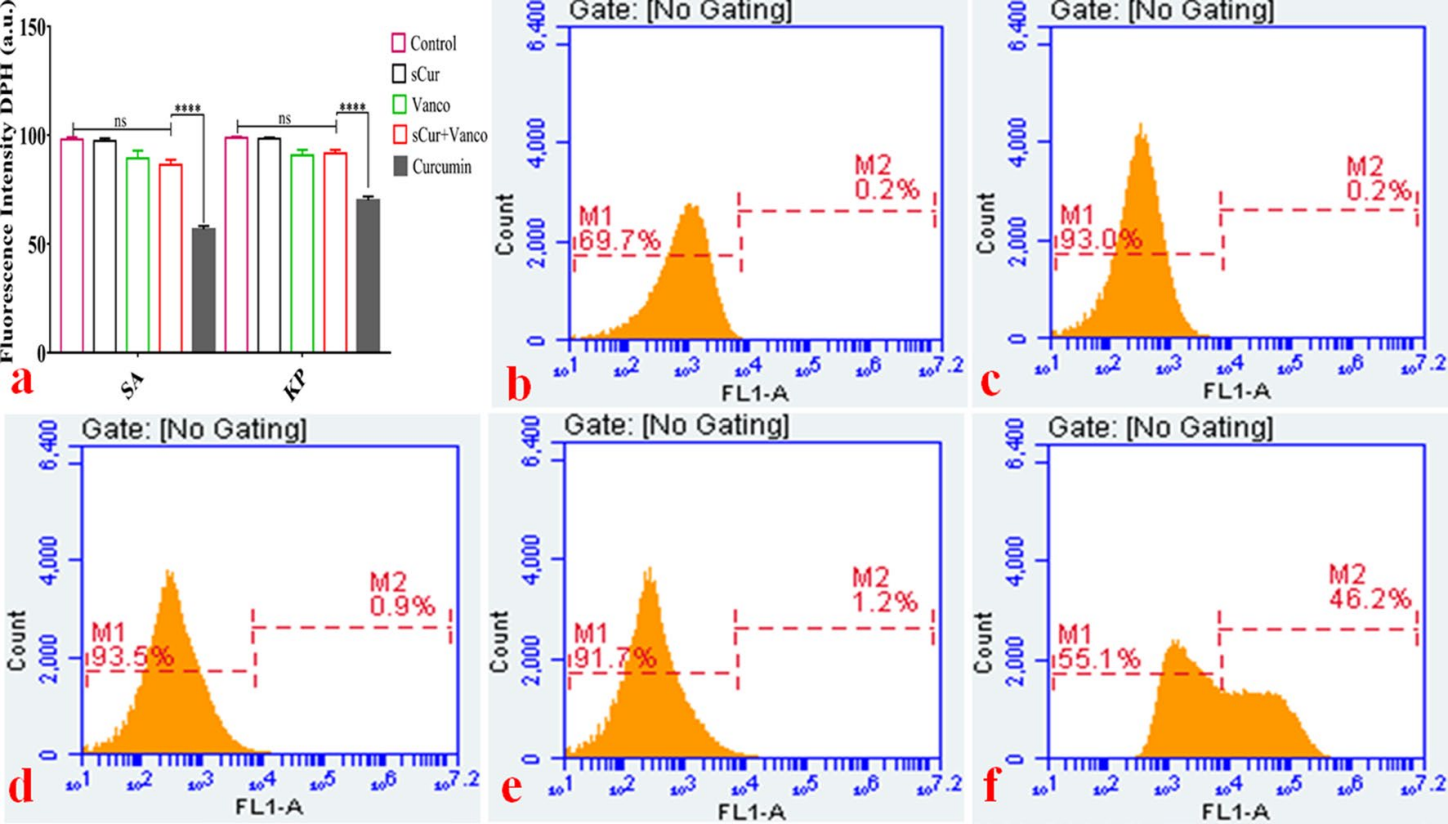
(**a**)* S*teady state 1, 6-diphenyl-1, 3, 5-hexatriene (DPH) based fluorimetry assay. The incubation of *Klebsiella pneumoniae* and methicillin resistant *Staphylococcus aureus* with MIC concentration of soluble curcumin, vancomycin and the combination of both (synergistic MIC concentration) for 120 min showed a varied extent of decrease in DPH fluorescence intensity in comparison to the untreated control as well as native curcumin. (**b**–**f**) Flow cytometry analyses of *Klebsiella pneumoniae* with DPH to explore the changes in its membrane dynamics in the groups treated with discrete sets of compounds and their combination. The symbols, pattern and colours used here remained the same for every panel. Total 1,000,000 cells were taken into account for each analysis. (**b**) Histograms of untreated logarithmic *Klebsiella* cells labeled with DPH. (**c**) Histograms of soluble curcumin (8 µg/ml) treated *Klebsiella* isolates labeled with DPH. Although there is minor increase in the intensity, there is no right shifting of the population that indicates the negligible acyl shifting/ change in membrane dynamics. (**d**) Histograms of vancomycin (512 µg/ml) treated *Klebsiella* isolates labeled with DPH. There is minor increase in the intensity compared to the untreated and mild right shifting of the population, indicating the negligible acyl shifting, or the change in membrane dynamics. (**e**) Histograms of soluble curcumin and vancomycin combination (8 µg/ml and 32 µg/ml (one fold higher concentration compared to the synergistic MIC) of the treated *Klebsiella* isolates labeled with DPH. There is minor increase in the intensity compared to the untreated and mild right shifting of the population (1.2%), indicating the negligible acyl shifting, or the change in membrane dynamics. (**f**) Histograms of native curcumin (512 µg/ml) treated *Klebsiella* isolates labeled with DPH. The panels show the significant right shifting indicating increase in cell population (46.2%) that had taken up DPH designating increased alteration in membrane lipids. (Graph was generated and analyzed using GraphPad 8.0 (San Diego, CA) while histograms were generated and analyzed using BD Accuri C6 Software).

The confocal microscopic studies have revealed the membrane leakage therefore; we investigated the sCur-mediated perturbations in membrane lipid dynamics by using DPH assay. DPH interacts with acylated lipids of the cell membrane, therefore intercalates in it, and hence fluoresce. However, if the membrane integrity is compromised, insertion of DPH into the membrane does not take place, and hence its fluorescence is also forfeited. We assessed it using both the steady-state fluorimetry assay and the flow cytometry. As we can see in Fig. 8a wherein the untreated control exhibited strong fluorescence intensity (Fluorescence unit ~ 10^2^ arbitrary units (a.u.) was set as threshold fluorescence). The incubation of *Klebsiella pneumoniae* with MIC concentration of sCur alone for 120 min showed insignificant decrease in DPH fluorescence intensity in comparison to the untreated control. As depicted in Fig. 8a, the DPH intensity was 93.44 ± 2.646, and 89.86 ± 2.07 a.u. for untreated *K. pneumoniae,* and MRSA clinical isolates. However, upon treatment with vancomycin, the fluorescence was found to be 89.21 ± 2.614 a.u, and 84.24 ± 1.875 a.u against *K. pneumoniae,* and MRSA isolates. Interestingly, upon exposure to the 8 µg/ml concentration of sCur (with inhibitory concentration of vancomycin (16 µg/ml) for 60 min, the DPH fluorescence intensity reduced to 89.17 ± 1.413 a.u and 83.46 ± 1.172 a.u. respectively against the said isolates. The Fig. 8a depicts the insignificant reduction in DPH fluorescence in comparison to the untreated control, negating the acyl shifting of membrane lipids.

We noted the same trend after the flow cytometry analysis. As shown in Fig. 8e, we confirmed that sCur and vancomycin combination (8 µg/ml and 16 µg/ml), caused negligible shift in the DPH fluorescence (and hence diminished right shifting, Fig. 8b–e), whereas incorporation of native curcumin (512 µg/ml) resulted in around 46% right shift indicating perturbed acyl groups (Fig. 8f). The results indicate that outer membrane (in Gram-negative isolates) and outer leaflet of the membrane (in Gram-positive isolate) remained unperturbed post exposure possibly owing to the pass through of sCur due to extended hydroxyl groups present. However, interestingly the combination of native curcumin and vancomycin had no significant action over membrane dynamics in the tested range (Supplementary information S5).

However, the confocal data was indicating the membrane leakage that compelled us to speculate that sCur acts on membranes indirectly by altering some other properties rather than by the direct binding or acyl shifting. The membranes are naturally predisposed to deformations to match the hydrophobicity of lipid bilayer and the anchoring interactions of the integral proteins that demands energetic forfeit. This status quo is utilized in this study. Possibly, the sCur altered its hydrophobic width. Recently, Ingolfsson et al*.* measured the effects of curcumin on the activity of gramicidin channels and reported that the addition of native curcumin increased both gramicidin channel lifetimes and their appearance rates^26^. From this, we clearly make out that curcumin has decreased the energetic penalty of the bilayer distortions.

### Membrane depolarization assay

**Figure 9:**
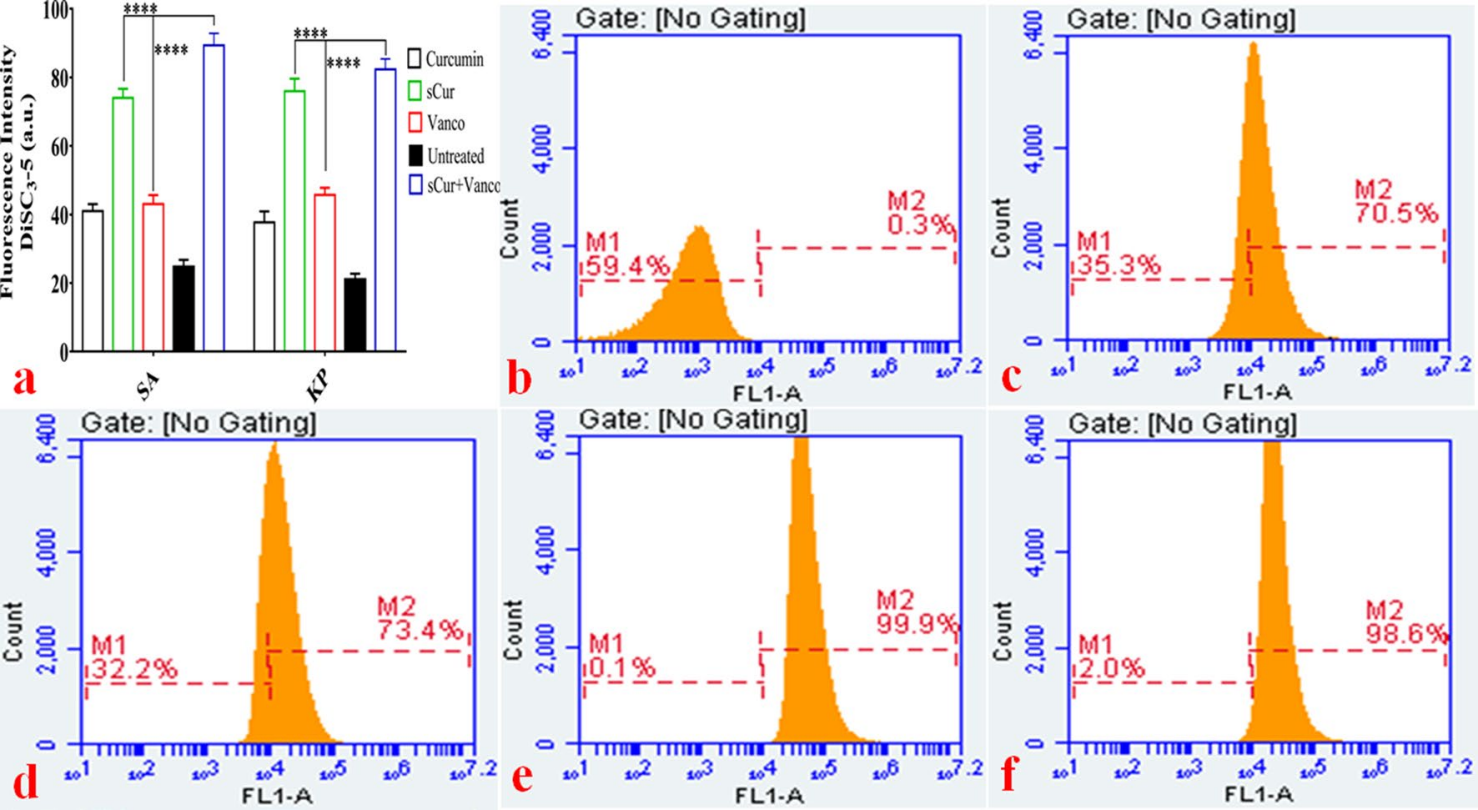
(**a**) *S*teady state 3, 3′-dipropylthiadicarbocyanine iodide based fluorimetry assay of *Klebsiella pneumoniae* and *Staphylococcus aureus*. The fluorescence intensity is function of leaked [DiSC_3_-5]. Therefore, the untreated control cells produce a low signal intensity and whereas the depolarized cells produce a high signal intensity. (**b**–**f**) Flow cytometry analysis of *Klebsiella pneumoniae* with membrane potential-sensitive dye, DiSC_3_-5 to explore the alterations in its membrane potential. (**b**) Histogram of untreated logarithmic phase *Klebsiella* cells labeled with DiSC_3_-5. (**c**) Histograms of native curcumin (512 µg/ml) treated *Klebsiella* isolates labeled with DiSC_3_-5. The panels show the increase in cell population (70.5%) that had taken up DiSC_3_-5 with increased intensity signaling the membrane depolarization. (**d**) Histograms of soluble curcumin (32 µg/ml) treated *Klebsiella* isolates labeled with DiSC_3_-5. The panels show the increase in cell population (73.4%) that had taken up DiSC_3_-5 denoting the membrane depolarization. (**e**) Histograms of the sCur-vancomycin treated *Klebsiella* isolates labeled with DiSC_3_-5. The panel demonstrates the absolute right shifting of the cell population (99.9%) that had taken up DiSC_3_-5 indicating the depolarization of nearly all the cells. (**f**) Histograms of the CCCP treated (positive control) *Klebsiella* isolates labeled with DiSC_3_-5. The panel demonstrates the absolute right shifting of the cell population (98.6%) that had taken up DiSC_3_-5 indicating the depolarization of nearly all the cells [Graph was generated and analyzed using GraphPad 8.0 (San Diego, CA) while histograms were generated and analyzed using BD Accuri C6 Software].

The presence of membrane electric potential (− 100 to − 200 mV) is the hallmark of metabolically active bacteria with intact cytoplasmic membranes. When the magnitude of this membrane potential reduces, it is referred to as electrical depolarization whereas an increase in the magnitude of membrane potential is referred to as electrical hyperpolarization. Membrane potential is reduced to zero if the membrane ruptures and becomes leaky and the change is irreversible. To investigate if sCur in combination resulted in membrane depolarization or reduces it to zero, we performed steady-state fluorimetry and flow cytometry investigations against multi-drug resistant *K. pneumoniae* and *S. aureus* isolates using the membrane potential-sensitive dye, DiSC_3_-5. The fluorescence of the dye decreases as it partitions into the surface of polarized cells, however, membrane depolarization rules out its partitioning, and hence the dye is released into the growth medium and increase in fluorescence intensity is manifested. Therefore, the untreated control cells produce a low signal intensity whereas the depolarized and leaky cells produce a high signal intensity. In this study, the untreated control exhibited negligible fluorescence. However, the addition of 8 µg/ml sCur along with vancomycin (16 µg/ml) resulted in a significant increase in fluorescence, indicating the sCur mediated depolarization of the cell membrane. As depicted in Fig. 9a, the DiSC_3_-5 intensity was 18.67 ± 2.082 a.u. for untreated *K. pneumoniae* and *S. aureus* isolates. Interestingly, upon exposure to the said concentration of the combination for 120 min, the DiSC_3_-5 fluorescence intensified to 91.24 ± 2.613 a.u. However, upon treatment with vancomycin alone, the fluorescence intensity was found to be 32.07 ± 5.224 a.u (Fig. 9a).

The same vogue was manifested in flow cytometry analysis wherein, sCur and vancomycin alone have fostered membrane depolarization in 70.5% and 73.4% of the total bacterial cell population (Fig. 9c, d). Strikingly, exposure to the combination instantaneously increased the depolarized/ leaked cell population (0.3–99.9%) comparison to the untreated cells (Fig. 9e, b). The results are comparable to the CCCP treated cells (Fig. 9f). However, interestingly the combination of native curcumin and vancomycin had no significant action over membrane potential in the tested range (Supplementary information S6).

These results clearly demonstrate that sCur foster membrane depolarization and cell leakage. The growth rate analysis along with bliss modelling discloses the synergistic effects which share time-dependent correlation between membrane depolarization and bacterial deaths. The data further suggested that the entire population of *K. pneumoniae* was rather instantaneously depolarized, resulting in unimodal distribution unlike the previous quantum construct of curcumin reported previously where progressive depolarization was encountered and hence bimodal distribution was observed. The cell leakage may therefore be due to the membrane depolarization showing consonance with the results obtained by CLSM data.

### Reactive oxygen species generation

**Figure 10:**
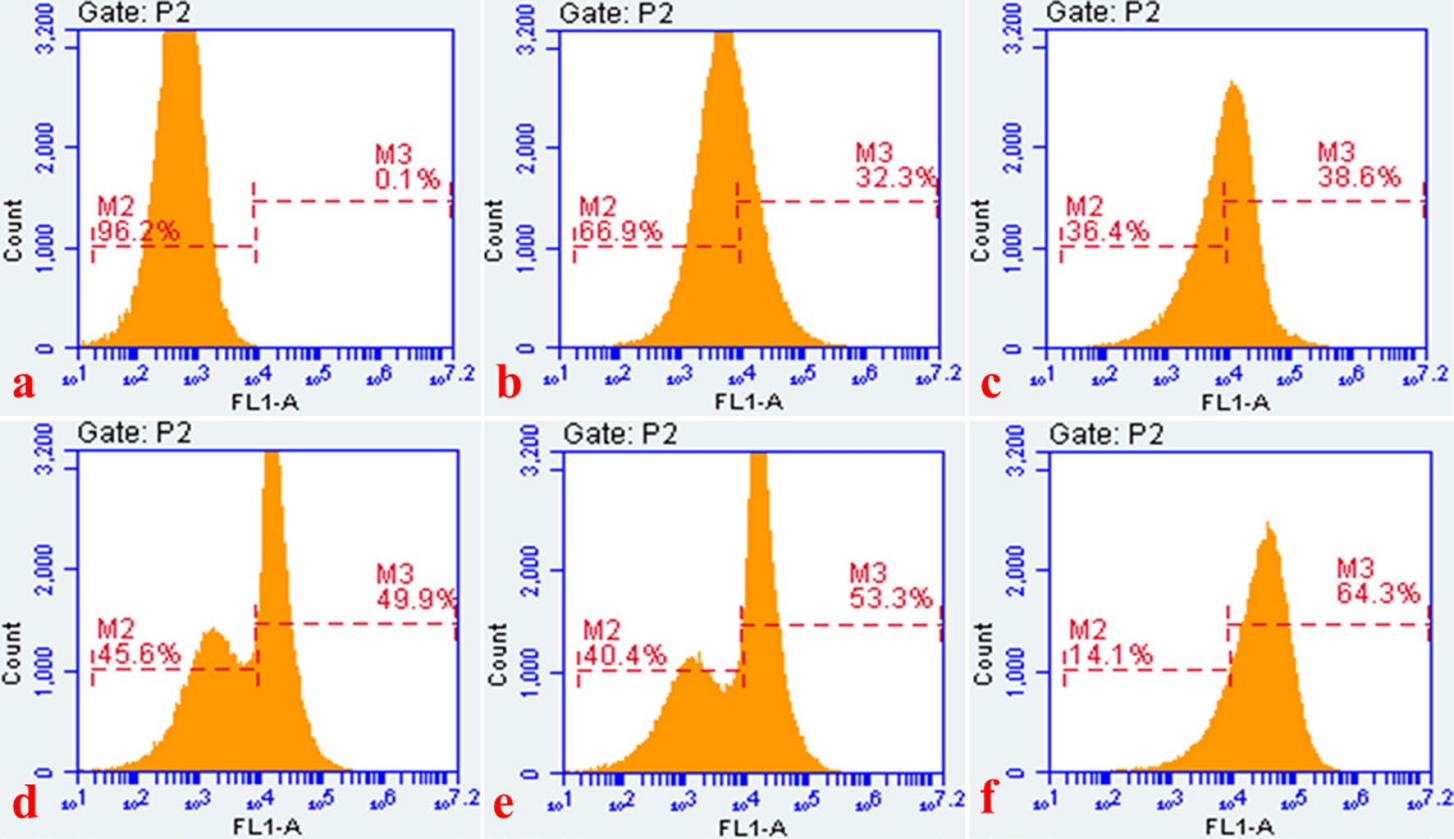
Time dependent flow cytometry analysis of *Klebsiella pneumoniae* with ROS-sensitive dye, DCFDA for ROS production analysis in the groups treated with soluble curcumin, vancomycin, and the combination of both. (**a**) Histogram of untreated log phase *Klebsiella* cells labeled with DCFDA. (**b**) Histogram of soluble curcumin treated *Klebsiella* isolates showing the increase in cell population (32.3%) that took up DCFDA showing ROS generation. (**c**) Histogram of vancomycin treated *Klebsiella* isolates showing the increase in cell population (38.6%) that had taken up DCFDA, showing the fair increase in ROS generation. (**d**) Histogram of the soluble curcumin-vancomycin treated *Klebsiella* isolates (for 2 h). The panel demonstrates the increase in cell population (49.9%) that had taken up DCFDA. Note the bimodal population distribution. (**e**) Histogram of the soluble curcumin-vancomycin treated *Klebsiella* isolates (for 4 h). The panel demonstrates the increase in cell population (53.3%) that had taken up DCFDA. Note the bimodal population distribution. (**f**) Histogram of the soluble curcumin-vancomycin treated *Klebsiella* isolates (for 6 h). The panel demonstrates the increase in cell population (64.3%) that had taken up DCFDA. Note the unimodal population distribution. (Histograms were generated and analyzed using BD Accuri C6 Software).

The bacterial isolates exposed to the sCur, vancomycin and the combination of the two, were evaluated for ROS generation by investigating with 2′, 7′-dichlorfluorescein-diacetate (DCFH-DA) using flow cytometry. We observed significant differences in the ROS generation profiles of these compounds (Fig. 10a–f). The combination of sCur and vancomycin orchestrated a substantial increase in the DCF fluorescence compared to the individual treatments (Fig. 10d–f). The population shift was significant (64.3%) in the combination treated group although it was time dependent. After 2 h of exposure 49.9% cells exhibited the ROS generation while the gross ROS generating population increased to 53.3% after 4 h showing bimodal distribution (Fig. 10d, e). However, after 6 h of treatment unimodal distribution was noted with 64.3% cells producing ROS. Conversely, sCur and vancomycin alone also triggered ROS generation compared to the untreated control as manifested by 32.3% and 38.6% shift in population post exposure with unimodal distribution (Fig. 10b, c). This showed that the combination has engendered significantly higher ROS in comparison to the individual treatments.

In addition to the said DCF fluorescence measurements as a marker of ROS, we specifically looked for the production of hydroxyl radicals (OH^•^) post treatment of the combination.

### Hydroxyl free radical estimation

**Figure 11:**
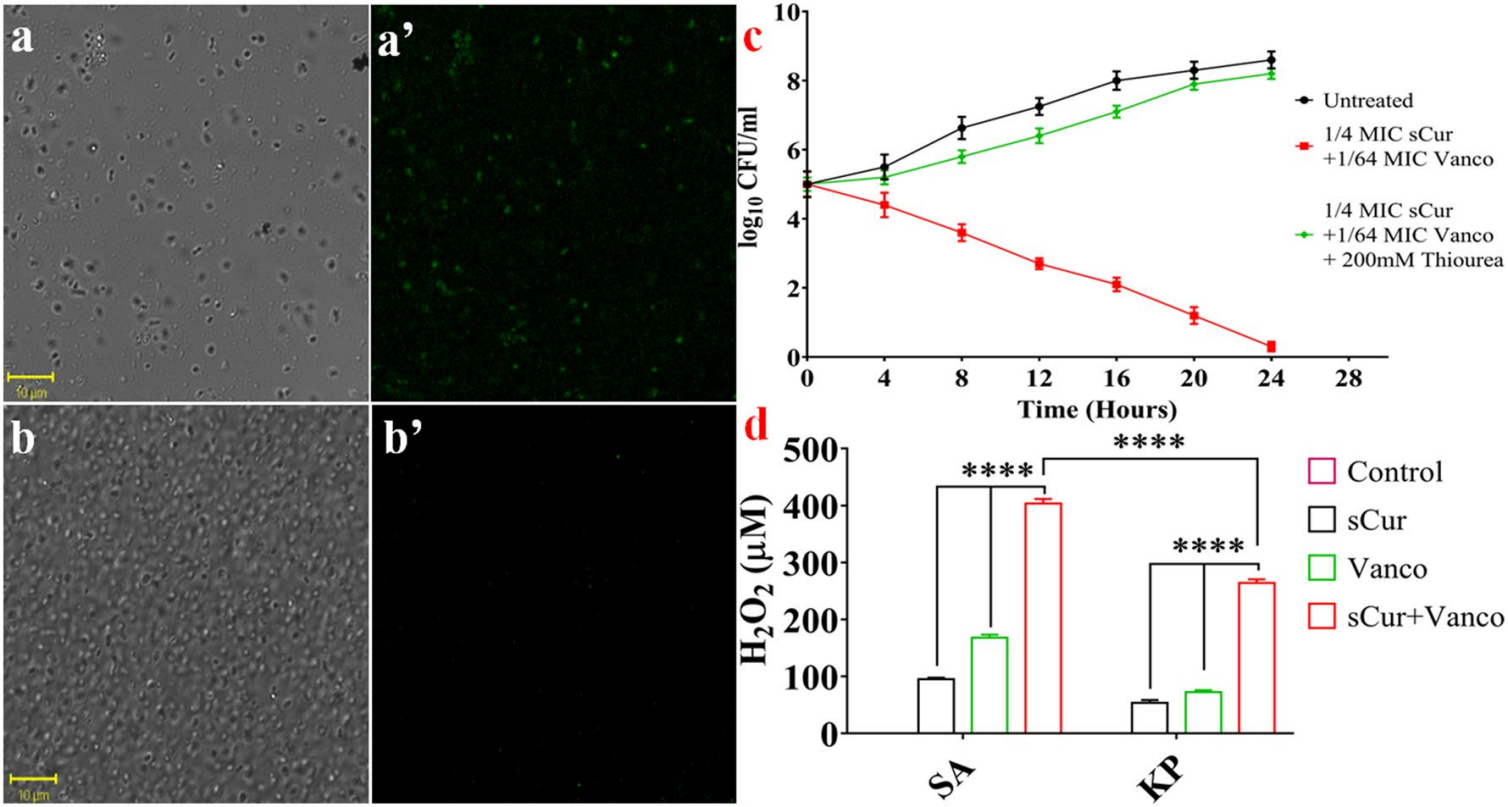
(**a**–**a’**) Bright field and fluorescence microscopy of HPF-stained treated cells with combination of soluble curcumin and vancomycin shows significant increase in the green fluorescent signals indicating significant increase in OH^•^ production. (**b**–**b**’) The loss of green fluorescent signals after addition of 200 mM thiourea, an ROS scavenger, depicts the reduction of sCur-vancomycin induced bacterial cell deaths. (**c**) Relative survival in terms of log_10_ CFU/ml change of *K. pneumoniae* with and without thiourea supplementation (**d**) Semi-quantitative estimation of H_2_O_2_ production under different conditions [Graphs were generated and analyzed using GraphPad 8.0 (San Diego, CA)].

We estimated hydroxyl radicals in untreated *S. aureus* and *K. pneumoniae* cells and in cells treated with sCur, vancomycin, and the combination of sCur and vancomycin for one hour, using 3′-(p-hydroxyphenyl) fluorescein (HPF) dye. The sCur-treated cells exhibited increases in fluorescence compared to untreated cells, indicating its role in OH^•^ production. However, vancomycin treated cells exhibited fair increase in the fluorescence, indicating good amount of OH^•^ production. However, the combination of sCur and vancomycin fostered the potential increase in the fluorescence, indicating significantly augmented OH^•^ production (Fig. 11a, a’).

Moreover, reducing ROS through the addition of 200 mM thiourea,  a known ROS scavenger, reduced this sCur-vancomycin induced bacterial cell death, confirming that ROS production may be critical for the observed bactericidal activity (Fig. 11b, c). The increase in cell population after administration of thiourea can be seen in DIC panel (Fig. 11b’).

### Hydrogen peroxide estimation

The data was further validated utilizing H_2_O_2_ estimation by semi-quantifying its production level post-exposure by Amplex Red. Astoundingly, the elevated H_2_O_2_ production was found to be 397 and 243 times higher than the untreated control in *S. aureus* and *K. pneumoniae* respectively (Fig. 11d)*.* Reports suggest that antibiotics kill by altering physiology by perturbing redox systems. Moreover, our results showed that 8 µg/ml sCur and 16 µg/ml vancomycin in combination fostered significant H_2_O_2_ production (P = 0.00001) compared to the control. Although, the treatment augmented ROS in both *S. aureus* and *K. pneumoniae*, but the effect was more discernable in *S. aureus* (Fig. 11d). The ROS elevation caused by sCur and vancomycin described here may inhibit the sulfhydryl dependent enzymes that led to the augmented bacterial deaths.

### Iron detection ferene-S colorimetric assay

**Figure 12:**
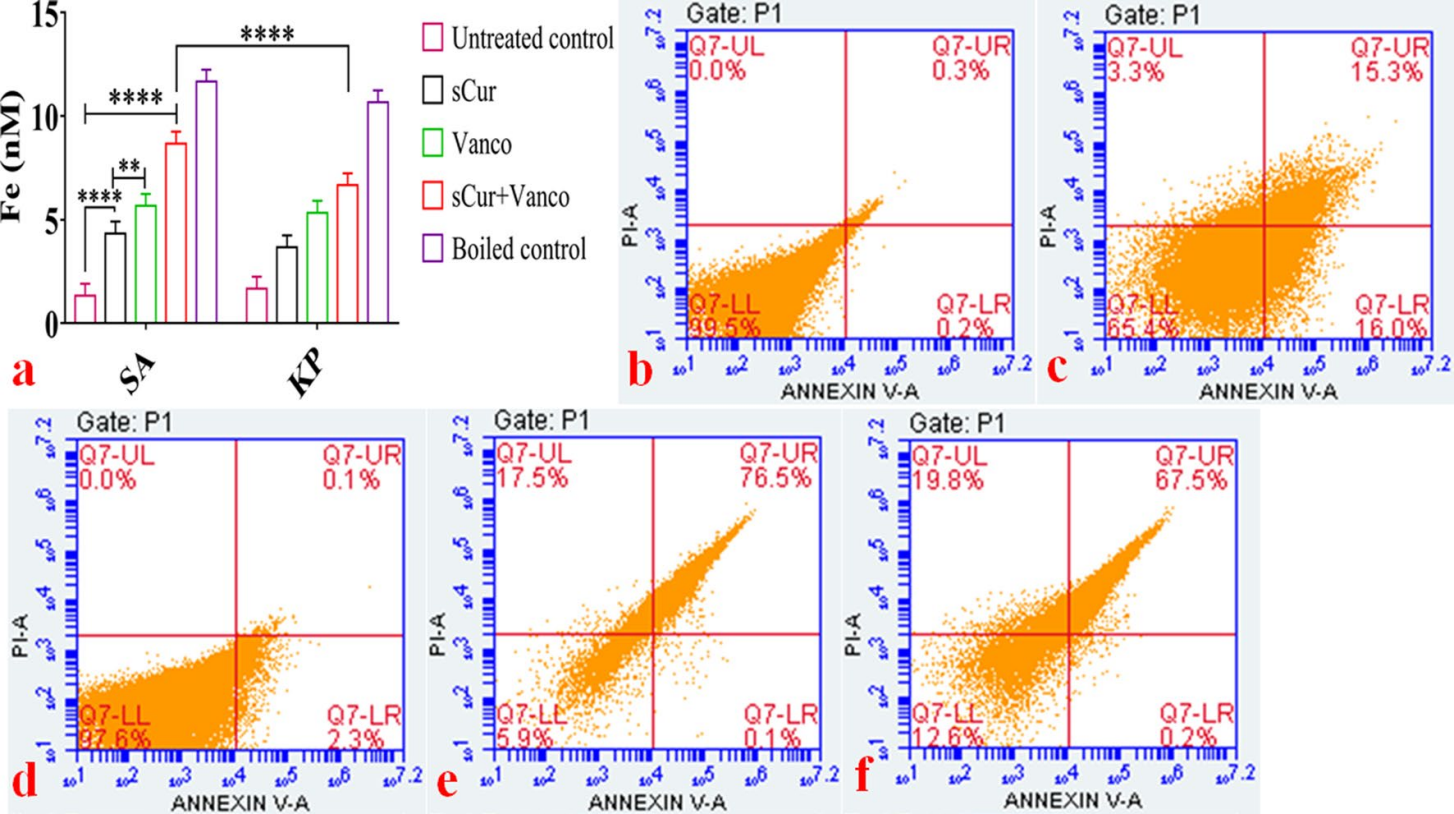
(**a**) Semi quantitative estimation of released Fe^2+^ by Ferene-S colorimetric assay. (**b**–**f**) Flow cytometry based evaluation of soluble curcumin-vancomycin mediated apoptosis-like phenomena utilizing Annexin-V and PI dual staining. (**b**) Histogram of untreated log phase *Klebsiella* cells labeled with Annexin V-PI. (**c**) Histogram of soluble curcumin treated log phase *Klebsiella* cells labeled with Annexin V-PI. (**d**) Histogram of vancomycin treated log phase *Klebsiella* cells labeled with Annexin V-PI. (**e**) Histogram of soluble curcumin-vancomycin treated log phase *Klebsiella* cells labeled with Annexin V-PI. (**f**) Histogram of native curcumin treated log phase *Klebsiella* cells labeled with Annexin V-PI. (Graph was generated and analyzed using GraphPad 8.0 (San Diego, CA) while histograms were generated and analyzed using BD Accuri C6 Software).

In general, the hydroxyl radicals are the product of Fenton’s reaction, where free iron plays a key role. Therefore, we measured the effect of sCur on iron homeostasis by testing its ability to disrupt Fe-S clusters (and cause release of Fe^2+^) by measuring Fe^2+^ concentrations using a colorimetric dye Ferene-S, in the sCur-treated *S. aureus and K. pneumoniae* cell lysates. After one hour of sCur treatment, test strains exhibited increased fluorescence compared to the untreated cells, indicating the perturbations in intracellular iron concentrations by affecting (either by breaking or by inactivating) the Fe-S clusters. For positive control, we heated the cell lysate to 90 °C to disrupt Fe-S clusters whereas the untreated lysate served as negative control. Interestingly, sCur-treated lysates showed significantly higher Fe^+2^ concentrations relative to the untreated lysate (p < 0.001), revealing that sCur directly interacts with and disrupts Fe-S clusters (Fig. 12a).

Together these findings indicate that sCur disturbs internal iron homeostasis by disrupting intracellular Fe-S clusters, which enable it to potentiate other bactericidal antibiotics that share a common mechanism of action involving the overproduction of ROS. The reasons for the “preferred” toxicity for the bacteria of the said combination might be due to the occurrence of only a Cys-containing ROS quenching armamentarium with a low ROS scavenging capacity.

### Annexin V-PI staining

Phosphatidyl serine is mainly localized in the inner leaflet of the membrane but during apoptosis and membrane disassembly, it is exposed on to the outer leaflet. As shown in the lower right (LR) quadrants of panels c–f of Fig. 12, PS exposure of 16, 2.3, 0.1 and 0.2% was elicited in *K. pneumoniae* cells treated with sCur (8 μg/ml), vancomycin (32 μg/ml), combination of sCur-vancomycin (8 μg/ml + 4 μg/ml), and native curcumin (256 µg/ml) respectively with loss of membrane integrity (PI stained in upper right quadrants) (Fig. 12). These data demonstrate that sCur could induce PS exposure on the outer leaflet with leaky membrane, the late-apoptosis hallmark, with necrosis (upper right quadrant). However, native curcumin along with vancomycin was found ineffective in the said context (8 µg/ml + 32 µg/ml) (Supplementary information 7).

### In vitro evaluation of cytotoxicity

#### Sulforhodamine B based cell proliferation assay

**Figure 13:**
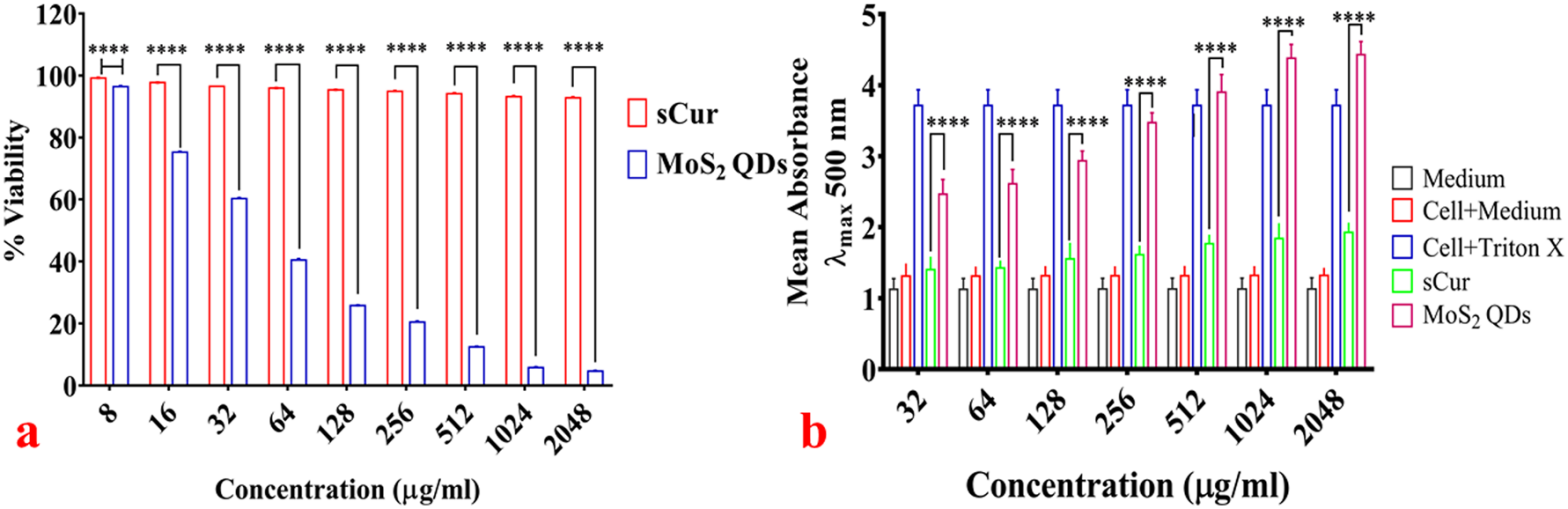
(**a**) Cell viability analysis using Sulforhodamine B (SRB) assay. The results of SRB assay corroborate well with the aforementioned assays. The cellular viability of HCT116 cells remained unperturbed even at highest treated concentration of soluble curcumin whereas MoS_2_ QDs had shown pronounced killing (< 5%) at 2048 µg/ml concentration. (**b**) Lactate dehydrogenase assay for cytotoxicity evaluation. The plot shows relative enzyme activity in terms of absorbance. The experiment had three controls parameters, 1. The medium alone; 2. Cells with the medium and 3. The cells treated with 2% Triton X 100. The minimal LDH activity was found in the group treated with soluble curcumin indicating minor cellular lysis while the MoS_2_ QDs treated group had shown dose dependent cell lysis with maximum LDH activity at the concentration of 2048 µg/ml. [Graphs were generated and analyzed using GraphPad 8.0 (San Diego, CA)].

The viability of HCT116 cells remained unaltered upon exposure to sCur in the tested range (8–2048 µg/ml) as insinuated from the fact that upon exposure to 2048 µg/ml, the cell viability remained 92.85% (almost unchanged). However, the MoS_2_ QDs affected the cell viability adversely in dose-dependent manner. For instance, 96.4% cells were found viable upon 8 µg/ml sCur exposure, while this viability declined to < 5% with escalation of dose to 2048 µg/ml. As seen, clearly in Fig. 13a, after 48 h, the viability remained unaltered compared to the MoS_2_ treated cells indicating its improved biocompatibility.

#### Membrane integrity analysis

Figure 13b unfolds the LDH activity (in terms of mean absorbance) in percentage after 48 h exposure of the HCT116 cells to the sCur and MoS_2_ QDs. At any given time, HCT116 cells incubated with sCur at various concentrations (32, 64, 128, 256, 512, 1,024, 2048 µg/ml) exhibited minimal LDH activities than the group treated with MoS_2_ QDs in the same concentration range. At the concentration of 2048 µg/ml, around 92.6% cytotoxicity was noted in the MoS_2_ QDs treated cells while on the same concentration no leakage was noted in sCur treated cells (Table 3). This indicates biocompatibility of sCur even at the higher doses, which were in consonance with the phase contrast micrographs (Fig. 3c–h).

**Table 3:** Lactate dehydrogenase cytotoxicity assay against HCT116 Cells.

Medium absorbance (λ_max_ 500)	Cell + medium absorbance (λ_max_ 500)	Cell + 2% Triton X 100 absorbance (λ_max_ 500)	Concentration (µg/ml)	sCur treated cells absorbance (λ_max_ 500)	MoS_2_ QDs treated cells absorbance (λ_max_ 500)	% cytotoxicity by MoS_2_ QDs
1.124 ± 0.125	1.318 ± 0.265	4.115 ± 0.304	32	1.403 ± 0.162	2.459 ± 0.217	0.706
			64	1.453 ± 0.134	2.577 ± 0.221	6.096
			128	1.521 ± 0.113	2.904 ± 0.144	18.104
			256	1.648 ± 0.126	3.429 ± 0.246	38.031
			512	1.750 ± 0.105	3.984 ± 0.293	54.014
			1,024	1.832 ± 0.134	4.357 ± 0.287	71.784
			2048	1.902 ± 0.182	4.684 ± 0.261	92.641

### In vivo evaluation of cytotoxicity

#### Body and organ weights

We recorded the body weights of all the test rats before and after sCur and MoS_2_ QDs administration. Among sCur treated groups III-VI, no significant variations in the overall body weights, vital organs, and body mass ratio were observed however, the MoS_2_ QDs treated group II has shown reduction in all these said parameters compared to the untreated control group I (Supplementary information 3, Tables T1, T2).

#### Biochemical evaluation of serum

Toxicity triggered by sCur was further assessed in blood serum and results are summed up in Supplementary information 3; Table T3. Serum glucose concentration remained unaltered in all sCur treated groups as well as MoS_2_ QDs treated group. However, a sharp reduction in Hb level was manifested in MoS_2_ QDs treated group (group II) while it remained infrangible among sCur treated groups. In addition, insignificant increase in the alanine aminotransferase (ALT/GPT), and aspartate aminotransferase (AST/GOT) activities among sCur treated groups (group III-VI) was perceived, however, in the MoS_2_ QDs treated group II, the discernable increase in ALT and AST activities was documented. The same altered vogue was manifested in serum urea and creatinine (p ≤ 0.05) levels in MoS_2_ QDs treated group II while insignificant change was noted among sCur treated groups (III–VI).

Of note, decreased activities of serum SOD and catalase was found (p ≤ 0.05) in MoS_2_ QDs treated group as compared to the untreated and sCur treated groups where no significant changes in their activities were realized (Supplementary information 3, Table: T4, T5).

#### Haematological profiling

We documented the red blood cell count (RBCs), total hemoglobin (Hb), white blood cells count (WBCs), mean corpuscular hemoglobin concentration (MCHC), platelet count (PLT), mean corpuscular hemoglobin (MCH), mean corpuscular volume (MCV) and hematocrit value (HCT) among groups. These haematological parameters remained almost the same in all sCur treated groups as compared to the untreated group (Supplementary information 3; Table: T6). Interestingly, the MoS_2_ QDs treated groups exhibited altered haematological profile (Supplementary information 3; Table: T6). These differences may be the outcome of unwanted interactions between MoS_2_ QDs and the blood cells leading to the incitement of immunological processes like inflammation.

#### Analysis of oxidative stress status

We evaluated the anti-oxidative indicators in the vital organs like liver, and kidney to assess the oxidative stress post treatment. The respective tissue protein contents and the respective enzyme activities have been listed in Supplementary information 3; tables T4 and T5 respectively. The lipid peroxidation level was assessed by malondialdehyde assay (Supplementary information 3, Table T5). We noted insignificant difference in the lipid peroxidation profile of sCur treated groups (III-VI) compared to the untreated control group I. Conversely, in MoS_2_ QDs treated positive control group II, we documented a significant increase in the lipid peroxidation level (p < 0.05) compared to the untreated control group I. Of note, the superoxide dismutase (SOD) and the catalase activities (CAT) declined significantly in MoS_2_ treated group II (p ˂ 0.05) while in sCur treated groups (III-VI) activities of SOD and CAT remained stable/unaltered as compared to the control group I (p > 0.05) (Supplementary information 3, Table T5). The reduction in SOD and CAT activities in MoS_2_ QDs treated group clearly reveal the shoot up in ROS generation.

#### Histopathological evaluation of vital organs

**Figure 14:**
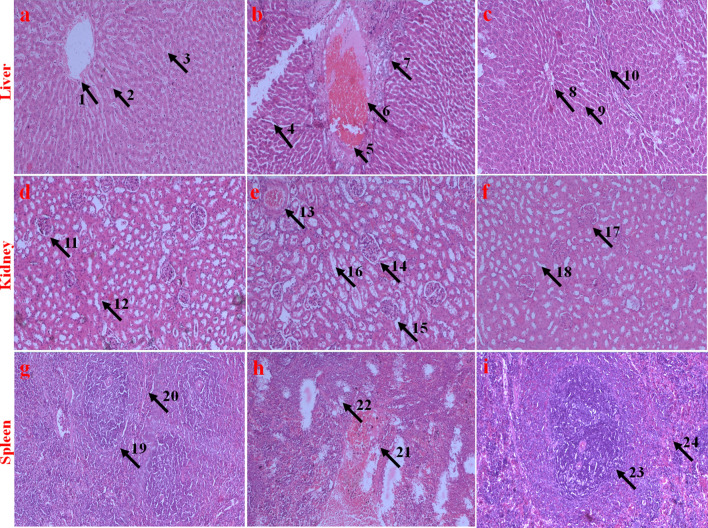
Histopathological evaluation (HE staining, 10 ×) of vital organs (Liver, Kidney, and spleen) of Charles Foster strain rats treated with highest dose of soluble curcumin and MoS_2_ QDs. (**a**), (**d**), (**g**) are liver, kidney and spleen sections of the untreated control group I; (**b**), (**e**), (**h**) are liver, kidney, and spleen sections of MoS_2_ QDs treated group II; and (**c**), (**f**), and (**i**) are liver, kidney and spleen sections of Group VI (highest dosed group). Labels (arrow and numeral) on the images indicate various regions of the tissues. (1) Undamaged central vein of group I. (2, 3) Compact and healthy hepatocytes of group I. (4) Damaged central vein of group II. (5) Dilated central vein of group II. (6,7) Degenerated, necrotized, vacuolated, and diffused hepatocytes of group II. (8) Undamaged central vein of group VI. (9, 10) Compact and healthy hepatocytes of group VI. (11) Healthy glomerulus and Bowman’s space of group I. (12) Healthy and undistorted loops of group I. (13) Necrotized glomerulus and Bowman’s space of group II. (14) Swollen glomerulus and dilated Bowman’s space of group II. (15) Regressed glomerulus and Bowman’s space of group II. (16) Distorted loops of group II. (17) Healthy glomerulus and Bowman’s space of group VI. (18) Healthy and undistorted loops of group VI. (19) Red pulp of the group I. (20) White pulp of the group I. (21) Regressed/necrotized red pulp of group II. (22) Regressed/ necrotized white pulp of group II. (23) Intact red pulp of group VI. (24) Intact white pulp of group VI.

We investigated the histopathological sections of vital organs like liver, kidney, and spleen to appraise the morphological changes post feeding. Figure 14 sums up the key histological findings of each organ in the respective test and control groups.

The liver of the untreated control group I (Fig. 14a) had the normal structure and compact arrangement of hepatocytes. We observed no obvious damage to the hepatocytes in sCur treated groups (III–VI) (Fig. 14c). However, we noticed apparent morphological changes in the MoS_2_ QDs treated group II liver tissues, like mild necrosis, hydropic degeneration, partial damage of the central vein, hepatocyte vacuolations, and even the degeneration of hepatocytes (Fig. 14b).

Likewise, the histopathological evaluations of kidney sections of highest dosed sCur treated group VI showed no discernable change compared to the untreated control group I (Fig. 14d, f). However, the histological study of the kidney of MoS_2_ QDs treated group II had mild nephrotoxicity, like inflated glomerulus and shrunken bowman's space (Fig. 14e). Interestingly, the same trend of toxicity was manifested in the spleen sections of the group II rats (Fig. 14h), whereas none of the sections of spleen in sCur treated groups showed any pathological alteration, as compared to the control group (Fig. 14i).

## Discussion

Over the last decade, synthetic experimentations and their theoretical modelling has fostered significant progress in our understanding of newer antimicrobials and their action against bacteria^27^. While previous works uncovered a range of non-trivial methods of synthesis with subsequent exploration of mere phenotypic effects over the ATCC control strains, our work rationalizes “Click” based synthesis of curcumin-sugar conjugate to potentiate the effects of cell wall, and gyrase based antibiotics^28,29^. Our work resolves a longstanding conundrum that curcumin cannot be used as the frontline therapeutic agent owing to its instability and aqueous insolubility^17,30–33^. In particular, our work documents the synthesis of completely water soluble curcumin with subsequent evaluation of its synergistic actions along with the otherwise tolerated antibiotics like ciprofloxacin, meropenem and vancomycin by the bacteria. We report herein that sCur amplifies the killing potential of tolerated antibiotics by increasing the membrane permeability and provides avidity for different cellular target(s), with unbearable ROS augmentation. We show that killing arises from its entraption along the width of the cell membrane that depolarizes the membrane leading to the ROS generation. This generated ROS triggers the release of Fe-S clusters and hence altering the iron as well as energy homeostasis, in which high-abundance hydroxide free radicals provide a buffer against ROS quenching enzymes. In this light, it seems plausible that bacteria fail to minimize the abundance of intracellular ROS as well as simultaneously imposed depolarization lead to membrane rupture, which ultimately  lead to cell deaths.

We synthesized bifunctional “clicked” galactose-curcumin conjugate that possesses two galactose moieties in accordance with our reported procedure in order to achieve our goal to synthesize a water-soluble, nontoxic, biocompatible derivative of curcumin, with antibacterial, anti-biofilm, and sensitizing activities at significantly lower concentrations compared to its native form. Click reaction is very regiospecific and therefore, in glycochemistry its use is in vogue^34^. We calculated the enhanced water solubility of sCur compared to the native curcumin by the UV–Vis experiment. From the spectrum and determined molar extinction coefficient, we measured solubility of sCur in water and found it almost ∼11, 000 times more soluble than curcumin in water.

By conducting phenotypic analyses, we herein document the sCur-mediated disruption of multiple bacterial cellular processes that result in the permeability alterations of the cell membrane and simultaneously generate excess ROS in both Gram-positive as well as Gram-negative bacterial isolates. Our work suggests that this multi-targeted antimicrobial mechanism of action is the result of innate thiophilic chemical properties of curcumin skeleton, which now became water-soluble. Through mechanistic explorations, we establish that sCur can potentiate the activity of a range of antibiotics, which ascertains its role as a potent antibiotic adjuvant.

More precisely, sCur imparted synergistic effects against MRSA and MDR *K. pneumoniae* when used in combination with beta-lactam, glycopeptide, and fluoroquinolone antibiotics. Clinically isolated these most difficult-to-treat Gram-negative pathogens are treated by last resort drugs carbapenems (like imipenem, meropenem) or the fourth-generation cephalosporins (cefepime etc.)^35^. Interestingly, the isolated strains were resistant to meropenem, imipenem, cefepime, and cefotaxime but were found sensitive to sCur added with ciprofloxacin, meropenem, and vancomycin in combination. We treated *E. coli, K. pneumoniae,* and *P. aeruginosa* with low doses of sCur (8 µg/ml) and vancomycin (16 µg/ml) individually and in combination, and found that the combination treatments resulted in significantly greater (p < 0.001) bacterial cell deaths relative to treatments with sCur or vancomycin alone. Using the Bliss Model, we determined the antimicrobial effects between sCur and vancomycin to be synergistic at all of the concentrations tested (Fig. 4). In this work, we studied the effects of sCur at sub inhibitory concentrations. The confocal and flow cytometry data unveil that even the sub inhibitory concentrations can foster moderate morphological changes in the membrane permeability, membrane polarization, and ROS generating status. Therefore, we used the activity of sCur to enhance the activity of other ROS generating antibiotics. We hypothesize that the rampant increases in ROS production had other ancillary effects like membrane leakage and perturbed iron homeostasis resulting in cell deaths; this is supported by our findings that incorporation of thiourea subdued the effects imparted by sCur-antibiotic combination and exhibited surged MIC indices. Although some investigators casted doubt on the role of the ROS in antibiotic-mediated bacterial deaths, in the current work we show, using various assays and experiments, that sCur treatment leads to ROS production in bacteria which was harnessed to enhance the killing efficacy of in use bactericidal antibiotics^36,37^. A plethora of literature documents very high MIC of native curcumin^24^. However, our work reveals that making curcumin water-soluble not only reduced its own MIC against the tested strains significantly, but also, astonishingly potentiated the action of conventional antibiotics by targeting multiple cellular sites, which may preclude the rapid emergence of resistance. Furthermore, our work indicates that even vancomycin in combination with sCur may become an alternative for treating Gram-negative infections as it permeabilize the outer membrane of Gram-negative bacteria. This may further facilitate us in repurposing of current drugs to enhance the present-day antibiotic arsenal.

We next examined whether the doses of sCur used in this study have a toxic effect on mammalian systems. We first assessed sCur toxicity in vitro, using the SRB cell viability assay on HCT116 cell lines. Our results showed that the cells exhibited no change in viability upon direct exposure to the sCur concentrations used even at the concentration 2048 µg/ml. While European food safety authority currently approves curcumin safe, we investigated the possibility of using sCur as sensitizer in combination therapies through other delivery routes (i.e., per oral administration)^38^. We next evaluated the effects of sCur on the blood chemistry and organ function of healthy, infection-free Charles Foster strain rats, by measuring key metabolite and enzyme concentrations. Rats treated with sCur showed normal kidney, liver and spleen functions. We observed insignificant increase in concentrations of the hepatic enzymes like alkaline phosphatase (ALP) and alanine aminotransferase (ALT), while total bilirubin remained unaltered. Histological evaluation of vital organs like liver, kidney and spleen endorsed the safety of the *per* oral use. While further studies are needed to fully determine the pharmacokinetic and pharmacodynamics profile, these in vivo results suggest that the sCur dosages used in this study are well tolerated by the test rats.

We are optimistic that future work from the fields of synthetic chemistry for the drug development and the nano sciences will tailor new modifications in sCur to develop more effective and broad range therapies. Moreover, given recent advances in synthetic sugar chemistry utilizing “click” and nanotechnology, we can envisage synthesizing soluble curcumin-metal complexes using other sugar combinations, which may be utilized in coating medical implants prone for secondary infections. In addition, even the antibiotic decorated quantum curcumin with some intelligent releasing material for controlled release of these curcumin-conjugates at the site of infection as and when required can be designed.

## Conclusion

In conclusion, we have successfully synthesized a “clicked” water-soluble (~ 11, 000 times more soluble than native curcumin) galactosylated curcumin with improved antibacterial, antibiofilm and antibiotic adjuvant activities in comparison to the native curcumin. In addition, we checked the biosafety profile of sCur by *per* oral administration in Charles Foster rats to ensure its biocompatibility. In all, the current study establishes the synthesis and application of galactose clicked curcumin as a new generation of therapeutic adjuvant, bestowing improved antibacterial activities against drug-resistant and high slime producing organisms without imparting cytotoxicity to the eukaryotic systems. It is envisioned that varying the sugar(s) in our prefabricated synthetic design, we can further fine-tune the sensitizing activities of sCur to curb the menace of multidrug resistance against a wide array of pathogens in amalgamation with in vogue therapeutic modalities.

Apart from its potential promise as antibiotic adjuvant, considerable improvements are needed to standardize the efficient downstream processing techniques for robust and scalable production, its storage conditions, and evaluations of pharmacokinetics and pharmacodynamics in humans are the obligatory issues needed to be dealt with before their clinical translation as antibiotic adjuvant.

## Methods

All the reactions were carried out at room temperature under ambient conditions. Thin layer chromatography (TLC) was performed on Merck 60 F254 silica gel, pre-coated on aluminum plates and revealed with either a UV lamp (λ_max_ ~ 254 nm) or by spraying with methanolic-H_2_SO_4_ solution with subsequent charring by heating it at 100 °C. NMR spectra were obtained on a JEOL AL500 FT-NMR spectrometer (500 MHz for ^1^H NMR and 75 MHz for ^13^C NMR) in CDCl_3_. Chemical shifts are given in ppm downfield from internal TMS and *J* values in Hz. Column chromatography was carried out on silica gel 234–400 mesh, E-Merck using n-hexane and ethyl acetate as the eluent.

### Synthesis of acetylated Galactose (2)

A suspension of galactose (5.0 g, 27.75 mmol) in acetic anhydride (25.0 ml) was taken in round bottom flask. Iodine (0.250 g, 1.97 mmol) was added to the suspension and stirred for 1 h, was monitored by TLC and after completion DCM (100.0 ml) was added to the solution of the reaction mixture. Mixture was washed successively with hypo solution and aqueous Na_2_CO_3,_ two times. Organic layer was collected and evaporated to afford the pure product (10.3 g, 26.36 mmol 95%) without column chromatography.

### Synthesis of galactose azide (2, 3, 4, 6-Tetra-*O-*acetyl-*β*-d-galactopyranosyl azide) (3)

Acetylated galactose (5.0 g, 12.8 mmol) was dissolved in dry DCM and temperature was maintained at 0 °C. In reaction mixture, HBr (12.0 ml) was added slowly under anhydrous condition and it was stirred for 3–4 h. at (0–10 °C). After completion of the reaction (monitored by TLC), the mixture was diluted with DCM and neutralized by NaHCO_3_ and extracted with dichloromethane. Organics were collected and dried over sodium sulphate and evaporated to obtain Per-*O*-acetylated galactose bromides. Further anomeric bromide (5.0 g, 12.15 mmol) without purification was dissolved in dry DMF were stirred with sodium azide (2.37 g, 3.0 equiv.) at 80 °C for 5 h. After completion of the reaction monitored by TLC, solvent was evaporated and extracted with EtOAc and washed with cold water, following evaporation of organic layer, column chromatography was done to obtain the pure product (3.38 g, 74%,).

White solid, 3.38 g, yield 74%; *R*_*f*_ = 0.4 (40% ethyl acetate/*n*-hexane); ^1^H NMR (500 MHz, CDCl_3_): δ 5.42 (d, *J* = 2.5 Hz, 1H), 5.18–5.14 (m, 1H), 5.06–5.03 (m, 1H), 4.62(d, J = 9.0 Hz, 1H), 4.20–4.14 (m, 2H), 4.05–4.02 (m, 1H), 2.17 (s, 3H), 2.09 (s, 3H), 2.06 (s, 3H), 1.99 (s, 3H); ^13^C NMR (125 MHz, CDCl_3_): δ 170.2, 169.9, 169.8,169.2, 88.1, 72.7, 70.5, 67.9, 66.7, 61.1 ppm.

### Curcumin di-alkyne (1*E*,6*E*)-1,7-bis(3-methoxy-4-(prop-2-yn-1-yloxy)phenyl)hepta-1,6-diene-3,5-dione (5)

Propargyl bromide (0.61 ml, 6.80 mmol) was added in fraction wise in the mixture of Curcumin (1 g, 2.7 mmol) and K_2_CO_3_ (0.76 g, 5.5 mmol) in the dry DMF under argon atmosphere and the mixture was stirred at room temperature for 48 h, Completion of the reaction was monitored by TLC and reaction mass was concentrated under reduced pressure to obtain crude. Purification of crude mass by flash chromatography (ethyl acetate: hexane) afforded curcumin derivative. Yield (58%); Orange red colour semi-solid, *R*_*f*_ = 0.5, (15% ethyl acetate/*n*-hexane).

Keto form: ^1^H NMR (500 MHz, CDCl_3_): δ 7.67 (d, *J* = 15.0 Hz, 2H), 7.08–7.06 (m, 4H), 6.96–6.92 (m, 4H), 6.60 (d, *J* = 15.0 Hz, 2H), 4.72 (d, *J* = 2.0 Hz, 4H), 3.83 (s, 6H), 2.45 (m, 2H); ^13^C NMR (75 MHz, CDCl_3_): δ 193.5, 149.8, 149.7, 145.7, 128.1, 128.0, 127.7, 123.6, 118.5, 113.5, 79.0, 76.4, 56.6 and 56.1 ppm. Raman Spectrum: 2,190 cm^−1^(Propargyl group). ESI–MS (m/z): [M+H]^+^ Calculated for C_27_H_24_O_6_ : 445.16, observed : 445.1

### Synthesis of curcumin clicked di acetylated galactose (6)

2, 3, 4, 6-Tetra-*O*-acetyl-*β*-d-galactopyranosyl azide (100 mg, 0.268 mmol) and Curcumin di-alkyne (35 μl, 0.321 mmol) were taken in dry CH_2_Cl_2_ (1 ml) in presence of CuSO_4_. 5H_2_O (2 mg, 2.7 μmol) and the mixture was stirred at room temperature for 5 h. Completion of the reaction was monitored by TLC and reaction mass was concentrated under reduced pressure to obtain crude. Purification of crude mass by flash chromatography (ethyl acetate: hexane) afforded triazole derivative. Yield (96%).

^1^*H* NMR (500 MHz, CDCl_3_): δ 7.94 (s, 1H), 7.92 (s, 1H), 7.17–6.91 (m, 4H), 6.73 (d, *J* = 8.0 Hz, 2H), 5.87–5.77 (m, 4H), 5.39 (d, *J* = 3.0 Hz, 1H), 5.28–5.24 (m, 2H), 4.99–4.90 (m, 5H), 4.67 (d, *J* = 8.0 Hz, 1H), 4.61–4.58 (m, 2H), 4.52–4.50 (d, *J* = 8.5 Hz, 1H), 4.45–4.41 (m, 1H), 4.28–4.20 (m, 2H), 3.92–3.83 (m, 5H), 3.76–3.65 (m, 6H), 3.54–3.48 (m, 2H), 2.21–2.06 (m, 24H); ^13^*C* NMR (75 MHz, CDCl_3_): δ 193.4, 183.5, 173.2, 172.7, 172.1, 171.2, 170.9, 170.0, 169.5, 152.6, 149.4, 148.0 144.5, 142.7, 139.2, 127.3, 127.0, 126.3, 121.9, 119.3, 119.2, 116.7, 115.1, 114.6 , 114.4, 114.0, 90.8, 88.4, 88.1, 87.9, 75.9, 75.7, 75.5, 74.2, 73.1, 72.7, 72.4, 72.3, 72.0, 71.1, 70.3, 69.1, 68.5, 68.4, 68.2, 67.8, 62.5, 62.4, 60.6, 60.2, 20.88, 20.85, 20.78, 20.72, 20.66 and 20.61 ppm. I.R. (cm ^-1^) 3,439.47, 2,922.21, 2,852.07, 1751.96, 1635.3, 1596.12, 1512.26, 1,464.79, (ESI–MS (m/z): [M + H]^+^ . Calculated for C_55_H_62_N_6_O_24_: 1,191.1, observed :1,192.

### Synthesis of De-*O*-acetylated curcumin digalactose (7)

Acetylated curcumin digalactose (50 mg) was dissolved in a mixture of Dry MeOH/dry THF/dry DCM (in the ratio 3:0.5:0.5 respectively and compound 6 2.5 × 10^–3^ M) and 20–30 µl solution of 1 M sodium methoxide was added. The reaction mixture was stirred for 4 h, then distilled water was mixed to completely solubilize the reaction mixture and the solution was made neutral (6–7 pH) by using ion exchange resin (Amberlite IR 120 H). Resin was filtered out and obtained solution was evaporated to achieve the compound **6** with 88% yield. Further, the purity of the compound was analysed by LC–MS.

^1^*H* NMR (500 MHz, CDCl_3_): δ 8.19 (s, 2H), 7.09–7.07 (m, 1H), 7.17–6.96 (m, 4H), 6.24 (d, *J* = 15.0 Hz, 1H), 5.56 (d, *J* = 5.5 Hz, 2H), 5.13–5.08 (m, 2H), 4.10–4.07 (m, 2H), 3.95–3.93 (m, 2H), 3.86–3.42 (m, 22H); ^13^*C* NMR (75 MHz, CDCl_3_): δ 171.0, 161.6, 161.5, 158.6, 158.3, 149.8, 149.1, 142.6, 140.8, 124.3, 124.1, 123.8, 122.7, 121.7, 114.2, 111.7, 100.1, 88.1, 88.0, 87.9, 78.3, 78.1, 72.9, 69.7, 68.6, 61.7, 61.6, 61.5, 60.8, 55.7 and 55.6 ppm. I.R. (cm^-1^): 3,417.67, 2,956.04, 2,920.02, 2,851.14, 1576.73, 1,540.41, 1,466.42. ESI–MS (m/z): [M+H]^+^ Calculated for C_39_H_46_N_6_O_16_: 856, observed : 856 and LC–MS: 854.

## Solubility comparison between curcumin and galactose conjugated curcumin

The dramatically augmented aqueous solubility of the galactose clicked curcumin was substantiated utilizing UV–Vis spectrometry. Briefly, 5 mg of sCur was vortex-mixed in 1 ml of MilliQ water in micro centrifuge tube to prepare the stock solution. For comparison, native curcumin (5 mg) was also vortex-mixed in 1 ml of MilliQ water in micro centrifuge tube. Both the samples were then centrifuged at 13,000 RPM for 5 min to remove undissolved part if any. Then, 100 µl of sCur was added to 900 µl of MilliQ water and mixed properly. The absorbance profile of the diluted sCur and native curcumin was documented.

### Biological investigations

#### Bacterial strains and culture conditions

Initially the effects were observed on the select control bacterial strains namely *Staphylococcus aureus* (ATCC 29,213), *Staphylococcus epidermidis* (ATCC 35,984), *Klebsiella pneumoniae* (ATCC 700,603), *Escherichia coli* (ATCC 25,922), and *Pseudomonas aeruginosa* (ATCC 25,619). However, the experiments were further extended to the multi-drug resistant clinical isolates of *Klebsiella pneumoniae* (Lab code: 10,894/2019), Methicillin-sensitive *Staphylococcus aureus* (Lab code: 2,862/2019, MSSA), Methicillin-resistant *Staphylococcus aureus* (MRSA, lab code: 2,859/2019), *Escherichia coli* (Lab code: 507/2019), and *Pseudomonas aeruginosa* (Lab code: 2,412/2019). The present study was approved by the Institutional Ethical Committee of Institute of Medical Sciences, Banaras Hindu University (Dean/2018/EC/594). The bacterial identification was based on conventional bacteriological techniques, such as colony morphology, gram staining, and different biochemical tests described earlier^39^. We defined multi-drug resistance in the present study as absolute resistance against at least five different drug-classes. Antibiotic susceptibility testing of the isolates was performed by modified Kirby–Bauer method in accordance with the Clinical and Laboratory Standards Institute guidelines 2019^40^. We used 14 antibiotic discs namely Ampicillin (10 µg), Amikacin (30 µg), Amoxicillin/clavulanate (20/10 µg), Levofloxacin (5 µg), Cephalexin (30 µg), Cefuroxime (30 µg), Gentamicin (120 µg), Ciprofloxacin (5 µg), Cefepime (30 µg), Co-trimoxazole (23.75/1.25 µg), Piperacillin & tazobactam (100/10 µg), Ertapenem (10 µg), Meropenem (10 µg), Imipenem (10 µg).

#### Minimum inhibitory concentration (MIC) determination

The minimum inhibitory concentration of sCur was determined by the broth microdilution method as described earlier with minor modifications^24^.

Briefly, *Staphylococcus aureus*, *Staphylococcus epidermidis*, *Klebsiella pneumoniae*, *Escherichia coli*, and *Pseudomonas aeruginosa* were grown in 10 ml BHI broth aerobically for 18 h. Bacterial culture (500 µl) was then diluted to 1.5 ml fresh BHI broth. The sCur stock solution (1,024 µg/ml) was used for the study. The stock was diluted in a series of two-fold dilutions ranging from 0.5 to 512 µg/ml in sterile BHI broth in microtiter wells. Each well of the 96-well microtiter plate was then inoculated with 190 µl of standardized cell suspension (10^5^ CFU/ml) and incubated at 37 °C for next 18 h along with the 20 µl sCur suspension. The MIC was defined as the lowest concentration of sCur at which no obvious growth was observed. Positive controls were having either ciprofloxacin or meropenem (for gram positive and gram negative respectively) while the sterile broth served as negative control and all the experiments were performed in triplicate.

#### Antibiofilm activity determination

The antibiofilm assay was performed utilizing semi-quantitative CV assay in 96-well tissue culture plate described previously^6,41^. Briefly, the overnight cultures of *Klebsiella pneumoniae*, *Escherichia coli*, and *Pseudomonas aeruginosa* were grown in Luria Bertani broth while *Staphylococcus aureus*, and *Staphylococcus epidermidis* were grown in BHI broth and diluted 1:1,000 with fresh broth. The 180 µl of each diluted bacterial suspension (0.5 McFarland’s) was doled out in flat-bottom polystyrene 96-well tissue culture plate and 20 µl of sCur solution (100 µg/ml) was added to each well. Wells without sCur were set up as negative controls while positive control wells contained curcumin-molybdenum hybrid quantum dots. Plates were incubated at 37 °C without shaking for 48 h to investigate its biofilm inhibitory potential. The biofilm inhibition was quantified by crystal violet (CV) assay as percentage reduction in its biomass content as described earlier.

$$\% \,\,Reduction = \frac{{\left( {Mean\,\, absorbance \,\,of \,\,the \,\,control - Mean\,\, absorbance \,\,of \,\,the \,\,test \,\,sample} \right)}}{{\left( {Mean\,\, absorbance \,\,of \,\,the \,\,control} \right)}} \times 100$$

#### Synergistic antibacterial activity of soluble curcumin in combination with antibiotics

We further accessed the synergism of soluble curcumin in combination with ciprofloxacin, vancomycin, and Meropenem using the classical checkerboard titration method by using 96-well polypropylene microtiter plate. Serial dilutions of with these antibiotics were mixed in cation-adjusted Muller Hinton broth. The inoculum was prepared from the overnight grown colonies on MHA. The final bacterial concentration after inoculation was 1.5 × 10^8^ CFU/ml. The MIC was determined after 24 h incubation at 37 °C. The MIC was defined as the lowest concentration of sCur alone or in combination with ciprofloxacin, vancomycin, and meropenem visibly inhibiting the bacterial growth.

#### Bacterial growth curve analysis

We spectrophotometrically monitored the effect of sCur on growth rates of the respective bacterial isolates both in the presence and absence of the drugs as described previously^19,42^. Briefly, overnight grown bacterial cells were allowed to grow in the fresh BHI broth to their early exponential phase. Once attained, the broth containing bacteria were inoculated into tissue culture plate with initial absorbance at λ_max_ 600 nm ~ 0.01. The change in absorbance of each well was then monitored after every 15 min for 4.5 h in both the situations. We determined the growth rate was by the slope of the linear part of the growth curve (R^2^, ≥ 0.98), determined for at least 5 data points of the semi-logarithmic plot of absorbance (ln [OD600]) v/s incubation time (in hours).

#### Quantifying synergy using the bliss model

Drug synergism was further calculated using the Bliss Independence Model described previously using the below mentioned formula:

$$S = \left( {{\raise0.7ex\hbox{${F xo}$} \!\mathord{\left/ {\vphantom {{F xo} {F oo}}}\right.\kern-\nulldelimiterspace} \!\lower0.7ex\hbox{${F oo}$}}} \right)\left( {{\raise0.7ex\hbox{${F yo}$} \!\mathord{\left/ {\vphantom {{F yo} {F oo}}}\right.\kern-\nulldelimiterspace} \!\lower0.7ex\hbox{${F oo}$}} } \right) - \left( {{\raise0.7ex\hbox{${F xy}$} \!\mathord{\left/ {\vphantom {{F xy} {F oo}}}\right.\kern-\nulldelimiterspace} \!\lower0.7ex\hbox{${F oo}$}}} \right)$$

where F_xy_ refers to the growth rate in the presence of the combination of test compounds at the concentration X, for sCur, and Y for the vancomycin^43^. Similarly, F_xo_ and F_yo_ refer to the growth rates in the presence of the individual test compounds say sCur and the other antibiotic at a concentration of X and Y, respectively. F_00_ refers to the growth rate in the absence of test compounds. S corresponds to the degree of synergy, a parameter that determines a synergistic interaction for positive values and an antagonistic interaction for negative ones. Growth rates at different time points are determined by calculating the slope of the growth curve being analyzed for 4.5 h as described above.

#### Confocal laser scanning microscopic evaluation of the synergistic antibacterial activity of soluble curcumin and vancomycin

For confocal analysis of the synergistic effects of sCur, we grew *K. pneumoniae* in chambered slides as described previously with modifications^42^. Briefly, we grew MRSA overnight and then diluted it 1:500 in fresh BHI broth to adjust its absorbance to 0.2 at λ_max_ 600 nm. Fifty microliters this diluted suspension was then dispensed into 8-well flat-bottom chambered slide containing 450 µl of BHI broth supplemented with 4% glucose and grown statically for 72 h at 37 °C. This was followed by time-dependent treatment with 20 µg/ml sCur and sub-inhibitory concentration of vancomycin (4 µg/ml). Prior to staining, we gently tapped the chambered slide to remove the residual broth and washed it thrice by phosphate buffer (pH 7.5) to remove the planktonic cells. We fixed the biofilm with 4% (v/v) paraformaldehyde for 30 min. The 10 µl of the reconstituted PI (10 µg/ml) was used applied directly to the top of the biofilms.

The Zeiss LSM 510 inverted confocal laser-scanning microscope (Carl Zeiss, Jena, Germany) was used to detect the green and red fluorescence from the dyes. Propidium iodide was excited with the HeNe2 530 nm laser and emission fluorescence was collected with the 620 nm filter. Images were obtained via a Plan-Neofluar 40X/1.3 oil objective with a z-step of 2.0 μm or 20 × objective with a z-step of 5.0 μm.

### Mechanistic insight

#### Study of changes in membrane dynamics

We utilized the previously described method^19^. Multidrug-resistant clinical isolates of *Staphylococcus aureus*, *Staphylococcus epidermidis*, *Klebsiella pneumoniae*, *Escherichia coli*, and *Pseudomonas aeruginosa* were treated with MIC concentration of sCur, vancomycin, and in combination (here data of only *Klebsiella pneumoniae* is shown). We incubated the treated cells for 120 min with shaking (100 rpm) at 37 °C, with subsequent harvesting. The pellet was re-suspended and re-pelleted. We fixed the pellet with 0.4% paraformaldehyde with subsequent resuspension in PBS. We later on incubated the re-suspended cells with 0.5 mM 1, 6-diphenyl-1, 3, 5-hexatriene (DPH) at 37 °C for 60 min, pelleted and washed thrice with PBS. The fluorescence intensity was monitored spectrophotometrically employing Synergy H1 Hybrid Multi-Mode Reader at 350 nm (excitation) and 425 nm (emission). Furthermore, the percentages of bacterial cells showing fluorescence response were measured using the flow cytometry (C6 BD Accuri, Becton–Dickinson, San Jose, CA, USA).

#### Membrane depolarization assay

The membrane potential-sensitive dye DiSC_3_-5 was used to measure plasma membrane depolarization in bacterial membranes as described earlier with modifications^44^. The isolates were cultured in BHI broth overnight with subsequent dilution to adjust the absorbance to 0.05 at λ_max_ 600. This was followed by the addition of 50 µl of MIC concentration sCur, vancomycin, and the combination for 2 h at 37 °C. This was further incubated for 20 min with 1 µM DiSC_3_-5. Negative control was without any drug while native curcumin treated bacterial lane served as positive control. The fluorescence was measured employing Synergy H1 Hybrid Multi-Mode Reader with excitation at 622 nm and emission at 670 nm. Besides, the percentages of bacterial cells showing fluorescence were measured using the flow cytometry (C6 BD Accuri, Becton–Dickinson, San Jose, CA, USA).

#### 2′, 7′-Dichlorfluorescein-diacetate (DCFH-DA) analysis for ROS production

Endogenous reactive oxygen species (ROS) production in bacteria after the exposure of sCur, vancomycin, and their combination for 48 h was monitored by flow cytometry using 2′, 7′-dichlorofluorescein-diacetate (DCFH-DA) as ROS marker described earlier^6^. After exposure for 2, 4 and 8 h to the sCur, vancomycin, and the combination, the bacterial cells were pelleted, washed thrice with phosphate-buffered saline (PBS, pH ~ 7.2) and then the cell density was adjusted to 10^7^/ml by suspending the cells in PBS. The resuspended cells were then incubated for 30 min with 5 μM DCFH-DA followed by analysis of ROS production on a BD Accuri C6 Flow cytometer. The data acquisition was performed with BD Accuri C6 software based on light-scatter and fluorescence signals resulting from 20 mW laser illumination at 488 nm. All the measurements were performed logarithmically. The assay was performed at a low sample rate (14-μl min^−1^). A total of 10^6^ events was taken into account for each sample.

### In vitro measurements of hydroxide free radical OH^•^

The fluorescent reporter dye 3′-(p-hydroxyphenyl) fluorescein (HPF) was used for hydroxide free radical detection as described previously^43^. Overnight cultures were diluted 1:250 with 25 ml of BHI broth in 250 ml flasks and incubated at 37 °C, with shaking (200 rpm). Cells were grown until they reached the OD600 ~ 0.3. Five hundred microliters sample was taken from the flasks and transferred to an 8-well plate for treatments. After 1 h of treatment with sCur and vancomycin, 200-μl sample was collected and pelleted by centrifugation at 10,000 rpm. The supernatant was exchanged with phosphate buffered saline supplemented with 5 mM HPF with incubation in the dark at room temperature for 15 min and re-pelleted by centrifugation at 10,000 rpm. The supernatant was removed and replaced with 1 × PBS for fluorimetry measurements.

#### H_2_O_2_ production assay

The H_2_O_2_ production was estimated by the method described previously^43^. Briefly, the bacterial cells were grown such that absorbance matched 0.4 at 600 nm in LB medium, and the bacterial cells were treated with 8 µg/ml vancomycin and 8 µg/ml sCur for 60 min. Cells were harvested by centrifugation at 6,000 rpm for 5 min and washed thoroughly thrice with PBS with subsequent sonication for 10 s. A total 50 µl of samples were incubated with 50 µM Amplex Red reagent, 0.1 U/ml HRP in 50 mM sodium phosphate buffer (pH 7.4), for 30 min at room temperature in dark with subsequent absorbance reading at 560 nm.

#### Iron detection Ferene-S colorimetric assay

The release of protein-bound iron in the bacterial cell lysate was measured using a Ferene-S assay reported earlier^43^. The lysate was prepared by first growing 150 ml of cells to an OD_600_ of 0.7. The cells were then lysed by sonication in 20 mM Tris/HCl pH ~ 7.2 buffer. The samples were centrifuged and the supernatants containing the cell lysates were collected. Lysates were treated either with heat (90 °C for 20 min) or with sCur, vancomycin, and their combination. 10 mM Ferene-S was added to each sample, and samples were then incubated at room temperature for 1 h. Absorbance at 593 nm was then measured with Synergy H1 Hybrid Multi-Mode Reader.

#### Annexin V/PI double staining

We utilized Annexin V–FITC apoptosis detection kit (Invitrogen, USA) to assess phosphatidyl serine (PS) exposure. For this, the MDR bacterial isolates were individually treated with sCur, vancomycin, and their combination and grown with shaking (120 rpm) for 2 h at 37 °C. The cells were pelleted (500 rpm for 15 min) and resuspended in PBS. We added 100 μl of Annexin V binding buffer, 5 μl of Annexin V–FITC, and 5 μl of PI to the cell suspensions and then, the mixtures were incubated at room temperature for 30 min. Post incubation, the cells were pelleted, and resuspended subsequently in 400 μl of Annexin V binding buffer, and analyzed using the C6 BD Accuri flow cytometer (Becton–Dickinson, San Jose, CA, USA).

### In vitro evaluation of cytotoxicity

The sCur and MoS_2_ QDs were further utilized for comparative evaluation toxicity. Prior to the experiment, MoS_2_ QDs were dispersed by ultra-sonication for 15 min with a power input of 750 W, frequency 10 kHz, and intensity 30 W/cm^2^ in pulse ratio on/off 50/10 (s/s) while sCur was vortex mixed.

#### Cell culture

HCT116 cells were propagated in Dulbecco’s modified Eagle’s medium containing 10% fetal bovine serum, 100 U/ml penicillin, and 100 mg/ml streptomycin in a 5% CO_2_ humidified atmosphere at 37 °C in a CO_2_ incubator. The cells were exposed to sCur and MoS_2_ QDs for 24 h. A 5 mg/ml stock solution of sCur, and MoS_2_ QDs were prepared, were stored as small aliquots at 4 °C, and diluted two folds in a different dose ranging from 2 to 2048 µg/ml in Dulbecco’s modified Eagle’s medium. Besides, the combination of sCur and vancomycin were also investigated for toxicity profiling as discussed above.

#### Cell proliferation assay

The sulforhodamine B (SRB) assay was performed as described previously^45^. The proliferating HCT116 cells were seeded into 96-well plates at a density of 5 × 10^3^ cells per well and allowed to adhere overnight. Twenty-four hours later cells were incubated for 48 h with a range of concentrations of sCur, and MoS_2_ quantum dots (2–2048 µg/ml). Cells were fixed with 50% (w/v) TCA and stained with 0.4% (w/v) sulforhodamine B (SRB) for 30 min before washing with 1% (v/v) acetic acid. SRB was solubilized with 10 mM Tris pH ~ 10.5, the absorbance read at 510 nm, and cell growth expressed as the percentage (%) of the growth of untreated cells.

#### Lactate dehydrogenase assay

The comparative cytotoxicity of sCur and MoS_2_ quantum dots were evaluated using HCT116 cells by measuring lactate dehydrogenase (LDH) activity as described earlier with modifications^19,46^. The two separate classes of test compounds were evaluated in concentration range 32, 64, 128, 256, 512, 1,024, and 2048 µg/ml. Briefly, the 12 h treated cells were spun at 400 g for 5 min and the growth medium was collected. The broth and the LDH reagent were incubated in the ratio of 2:1 for 30 min followed by the absorbance reading at 500 nm. For control, we used 2% Triton X treated cells. The extent of percentage (%) cytotoxicity was calculated as follows:

$$\frac{{\left( {Mean\,\, absorbance \,\,of \,\,Treated \,\,cells - Absorbance \,\,of \,\,Medium - Absorbance \,\,of \,\,Cell \,\,and \,\,Medium} \right)}}{{\left( {Absorbance \,\,of \,\,Triton \,\,X \,\,treated \,\,Cell - Absorbance \,\,of \,\,Cell \,\,and \,\,Medium} \right)}} \times 100$$

### In vivo evaluation of cytotoxicity

Ethical guidelines related to methods for using animals were strictly abided by for the care and use of Charles Foster strain rats, which were approved by the Institutional Animal Ethics Committee (IAEC), Banaras Hindu University, India (Ethical committee letter # No 2017/CAEC/720).

#### Animal

We followed the previously described method for the evaluation of in vivo cytotoxicity employing Charles foster strain rats. We maintained the thirty-six Charles Foster strain rats (male, 100–120 g) at 25 °C under a standard regimen of 12 h:12-h light–dark cycle and fed standard pellet diet. Prior to the start of the experiment, the animals were acclimatized to the experimental environment for 1 week.

Post acclimatization, we randomly divided rats into six groups such that each group contained six rats. One of the groups (Group I) was selected as the negative control (MiliQ water fed group), while another constituted the positive control (2048 µg/ml MoS_2_ QDs, Group II) and rest four groups (Group III–VI) were set as the experimental groups which were fed with 256, 512, 1,024, 2048 µg/ml sCur respectively.

After the first oral dose administration, general observations like changes in body weight, activity, and physical appearance were made in the first four hours. All the test rats were sacrificed to collect the whole blood for hematological and serum biochemical assays. Liver, kidney, and spleen were excised aseptically and weighed for histological and biochemical studies. Tissue homogenate was used for biochemical estimations of glucose, alanine aminotransferase (ALT), aspartate aminotransferase (AST), creatinine, urea, lipid peroxidation, SOD, and catalase (for detailed method, refer to supplementary information 3). We estimated SOD and catalase manually for serum as well. Blood parameters were also analyzed (see supplementary information 3).

#### Histopathological investigation

The excised tissues (liver, kidney, spleen) were washed with chilled normal saline (0.9% NaCl) and 20 mM EDTA and then immediately fixed in 10% formalin for 72 h. Subsequently, the tissues were stored in 70% ethanol, paraffin embedded, diced into 0.5 μm thicknesses blocks followed by mounting and staining with hematoxylin and eosin (H&E) for histopathological examination. Minimum five slides per sample were prepared (see supplementary information 3).

### Statistical analysis

The experiments were executed in triplicate, and results were averaged, and expressed as mean values with the corresponding standard deviations (SD). Statistical significance was analyzed by Two-way analysis of variance (ANOVA) applying Dunnett’s post hoc test. Besides, Student’s t-test (two-tailed, unequal variance), and Mann Whitney U test were also applied as and when required. Results of the animal studies were analyzed using two-way analysis of variance (ANOVA) for multiple comparisons followed by Newman-keul post hoc analysis. All the statistical calculations were done using GraphPad Prism version 7.0 (GraphPad Software, Inc., La Jolla, CA, USA). We considered P-value of < 0.05 as statistically significant.

## Supplementary information

Supplementary file 1
