# Supplementary material for: Making of water soluble curcumin to potentiate conventional antimicrobials by inducing apoptosis-like phenomena among drug-resistant bacteria
Source: Sci Rep. 2020 Aug 26;10:14204. doi: 10.1038/s41598-020-70921-2 (PMC7450046; doi:10.1038/s41598-020-70921-2)
Supplement: Supplementary file 1 [file 41598_2020_70921_MOESM1_ESM.pdf]

Supporting Information

Making of Water Soluble Curcumin to Potentiate Conventional Antimicrobials and Induce Apoptosis-like phenomena among Drug-Resistant Bacteria

Shivangi Yadav[1#], Ashish Kumar Singh[1#], Anand Kumar[2], Kavyanjali Sharma[3], Anoop Shyam Singh[2], Munesh Kumar Gupta[1], Vinod Kumar Tiwari[2*], Pradyot Prakash[1*]

[1]Bacterial Biofilm and Drug Resistance Research Laboratory, Department of Microbiology, Institute of Medical Sciences, Banaras Hindu University, Varanasi 221005, India.

[2]Department of Chemistry, Institute of Science, Banaras Hindu University, Varanasi 221005, India

[3]Department of Pathology, Institute of Medical Sciences, Banaras Hindu University, Varanasi 221005, India.

[#] Contributed Equally

[*] Corresponding Author

Emails of corresponding authors: pradyot_micro@bhu.ac.in, Tiwari_chem@yahoo.co.in

Table of Contents:

**S1.** ^{1}H and ^{13}C NMR spectrum of the synthesized compounds (**2-6**) (Supplementary information 1)	3-13
**S2.** IR spectra of the compounds 5 and 6 (Supplementary information 2)	14
**S3.** *In vivo* evaluation of cytotoxicity (Supplementary information 3)	15-16
**Table T1**: Body weight indices of control and test group rats	17
**Table T2:** Vital organ indices and Organ/body weight ratio of different groups on 15th day	18
**Table T3:** Kit based serological profiling of control and test group rats	19
**Table T4:** Protein estimation from liver, kidney, and serum among different groups of rats	20
**Table T5:** Activities of enzymes in liver and kidney tissues respectively (A) lipid peroxidation as the level of TBARS/ MDA concentration (B) activity of superoxide dismutase (C) activity of catalase in various groups of rats	21
**Table T6:** Automated hematological profiling of control and test group rats	22

**Table T7:** Modified Minimum inhibitory concentration of Vancomycin, Meropenem, and Ciprofloxacin upon administration with native curcumin (8 µg/ml) against MDR pathogens	23
**S4.** Confocal view of comaparative evaluation of curcumin and vancomycin combination on *Klebsiella pneumoniae* viability at different concentrations (Supplementary information 4)	24
**S5.** Flow cytometry analyses of *Klebsiella pneumoniae* with DPH to explore the changes in its membrane dynamics in the groups treated with discrete sets of compounds and their combination.	25
**S6.** Flow cytometry analyses of *Klebsiella pneumoniae* with $DiSC_3$-5 to explore the changes in its membrane potential in the groups treated with discrete sets of compounds and their combination.	26
**S7.** Flow cytometry analyses of *Klebsiella pneumoniae* with Annexin V-PI to explore the apoptosis like phenomenon in the groups treated with discrete sets of compounds and their combination.	27

^{1}H and ^{13}C NMR Spectrum of synthesized compunds

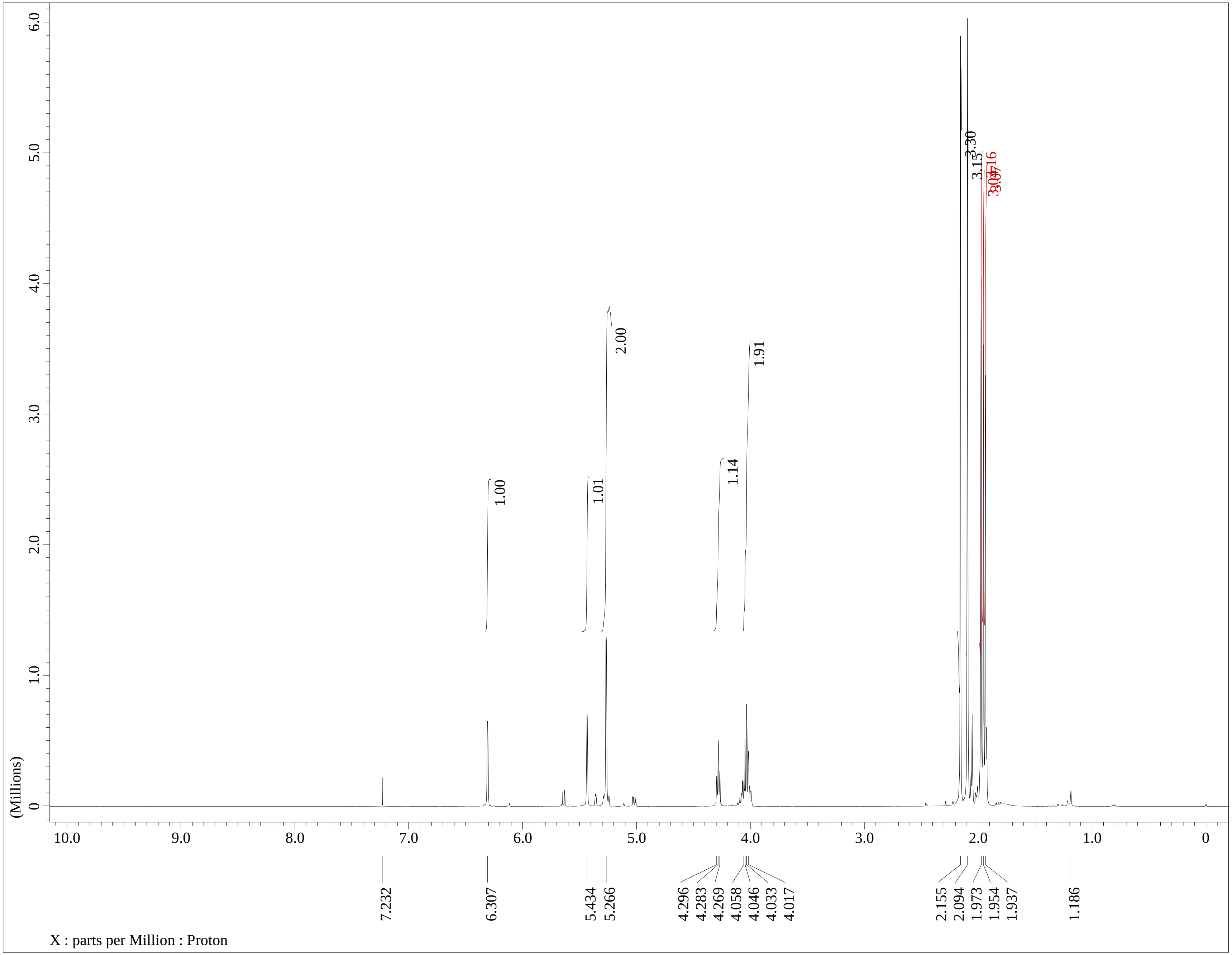

Spectra 1: ^{1}H NMR (500 MHz, $CDCl_3$) of compound **2**

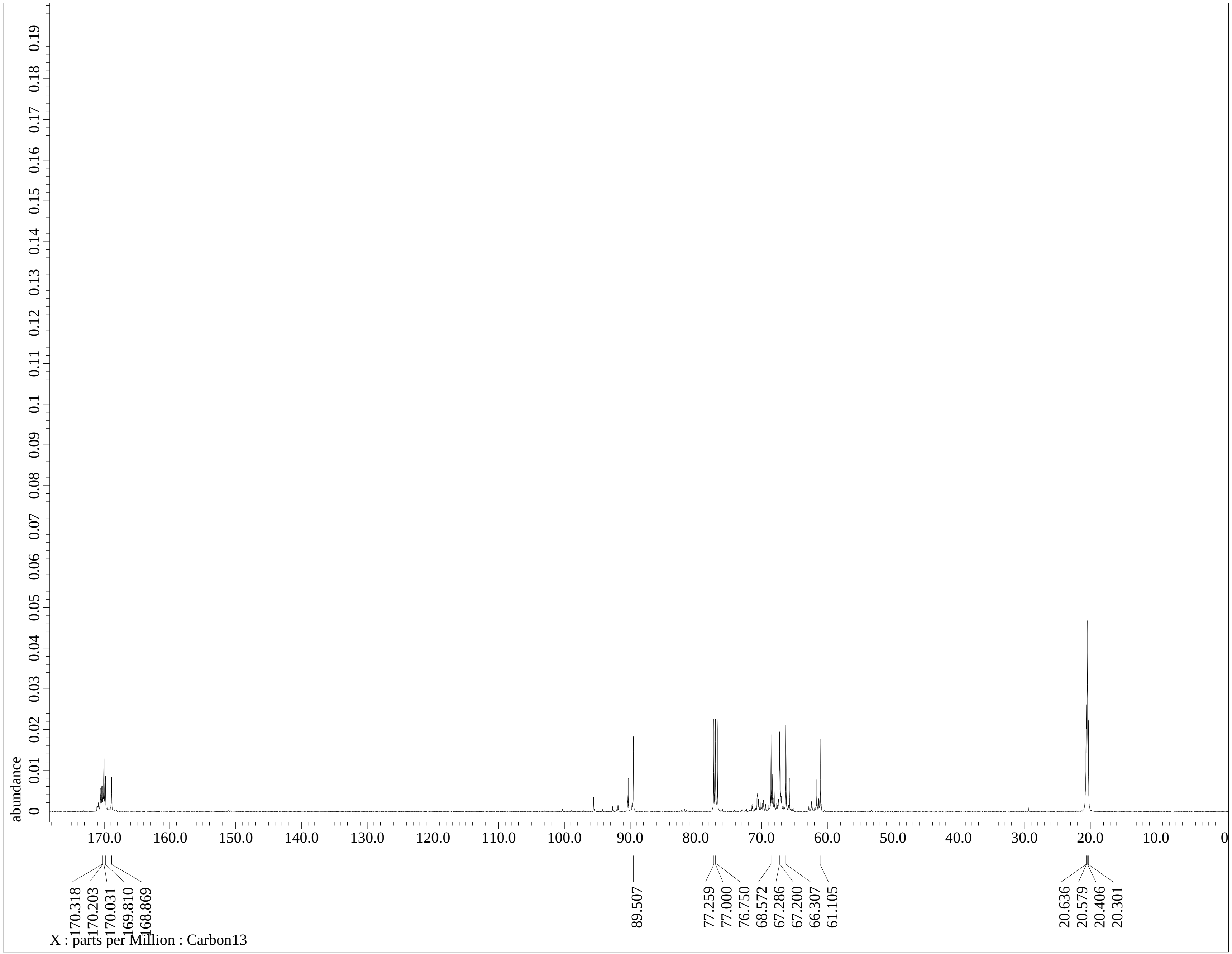

Spectra 2: ^{13}C NMR (125 MHz, $CDCl_3$) of compound **2**

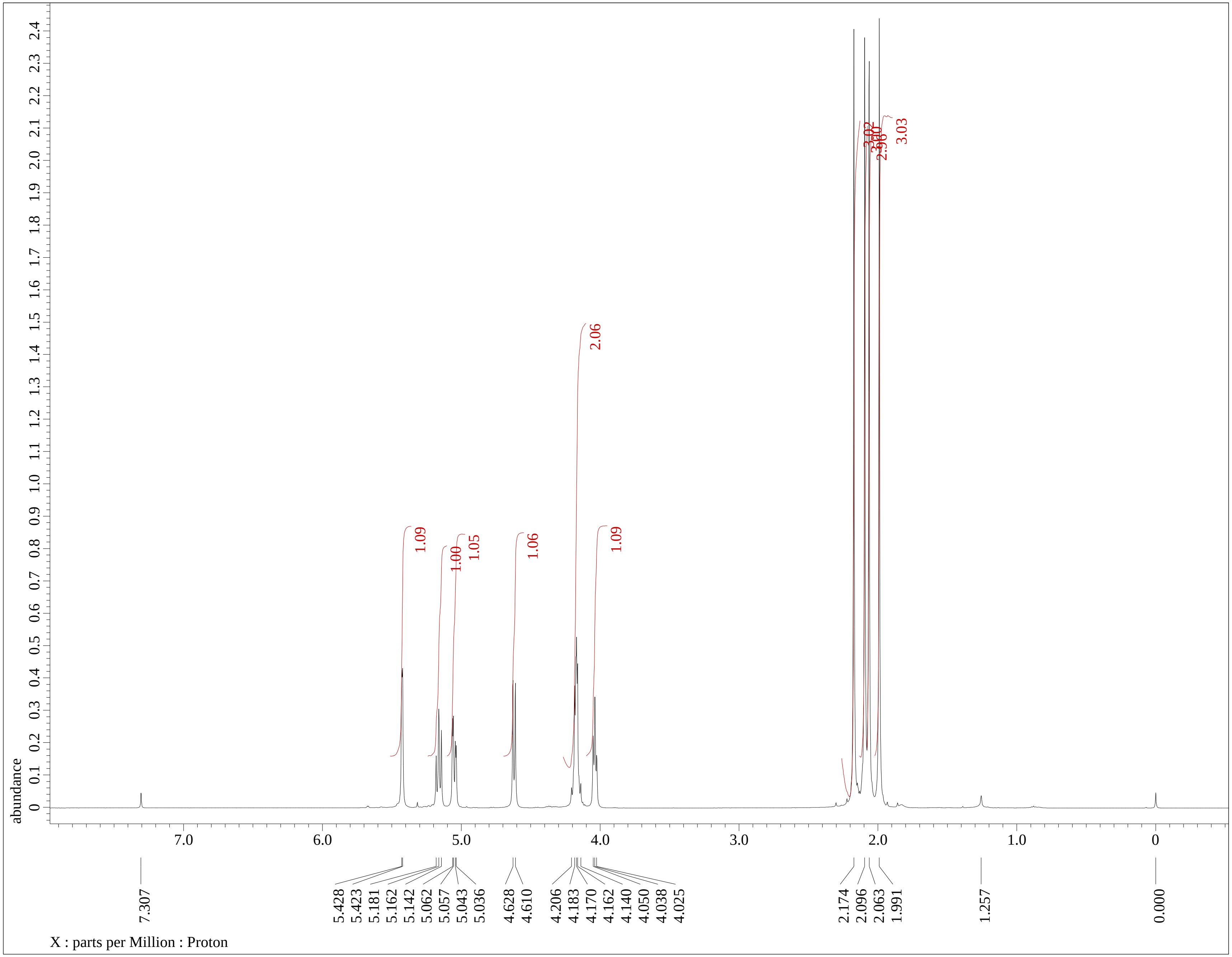

Spectra 3: ^{1}H NMR (500 MHz, $CDCl_3$) of compound **3**

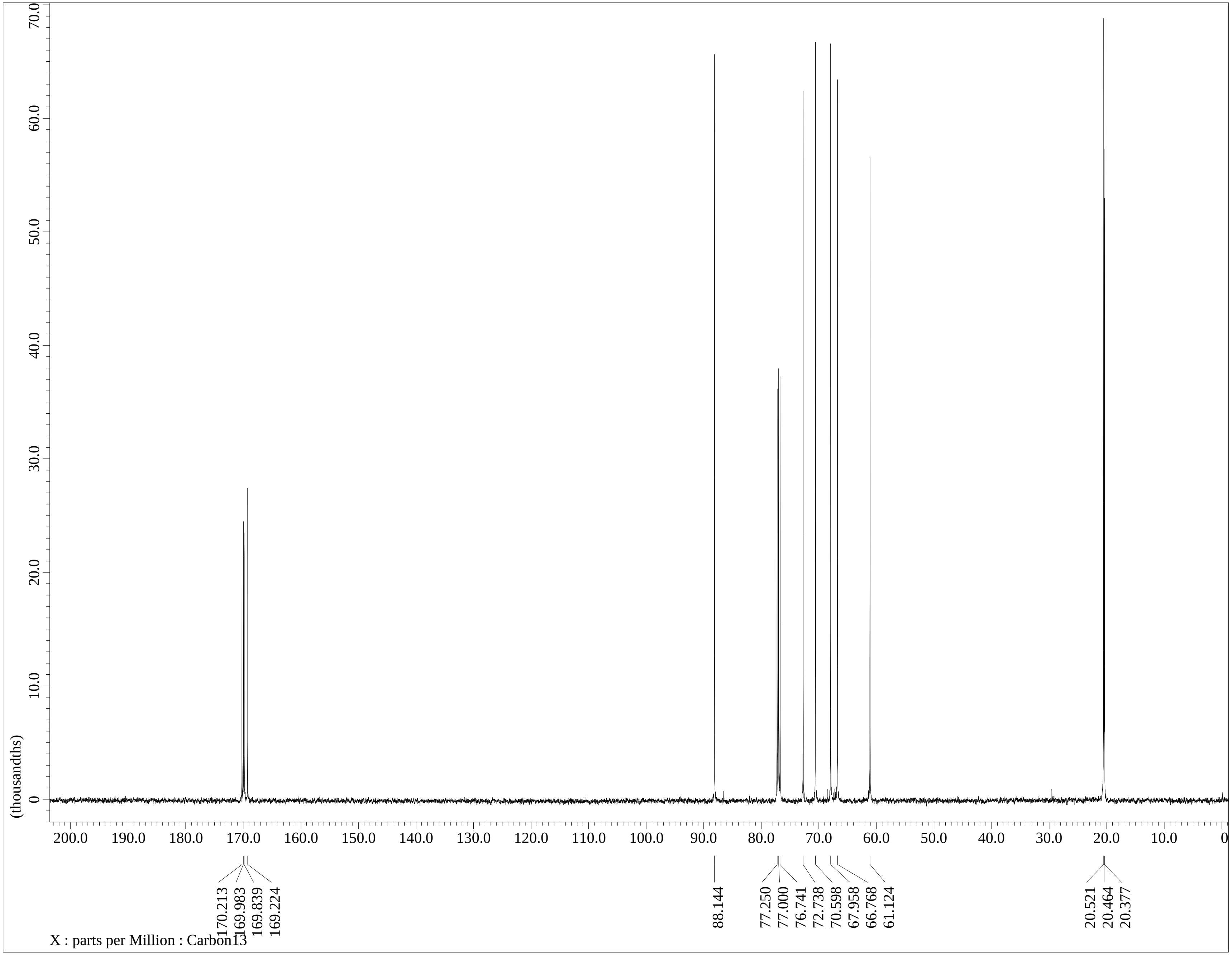

Spectra 4: ^{13}C NMR (125 MHz, $CDCl_3$) of compound **3**

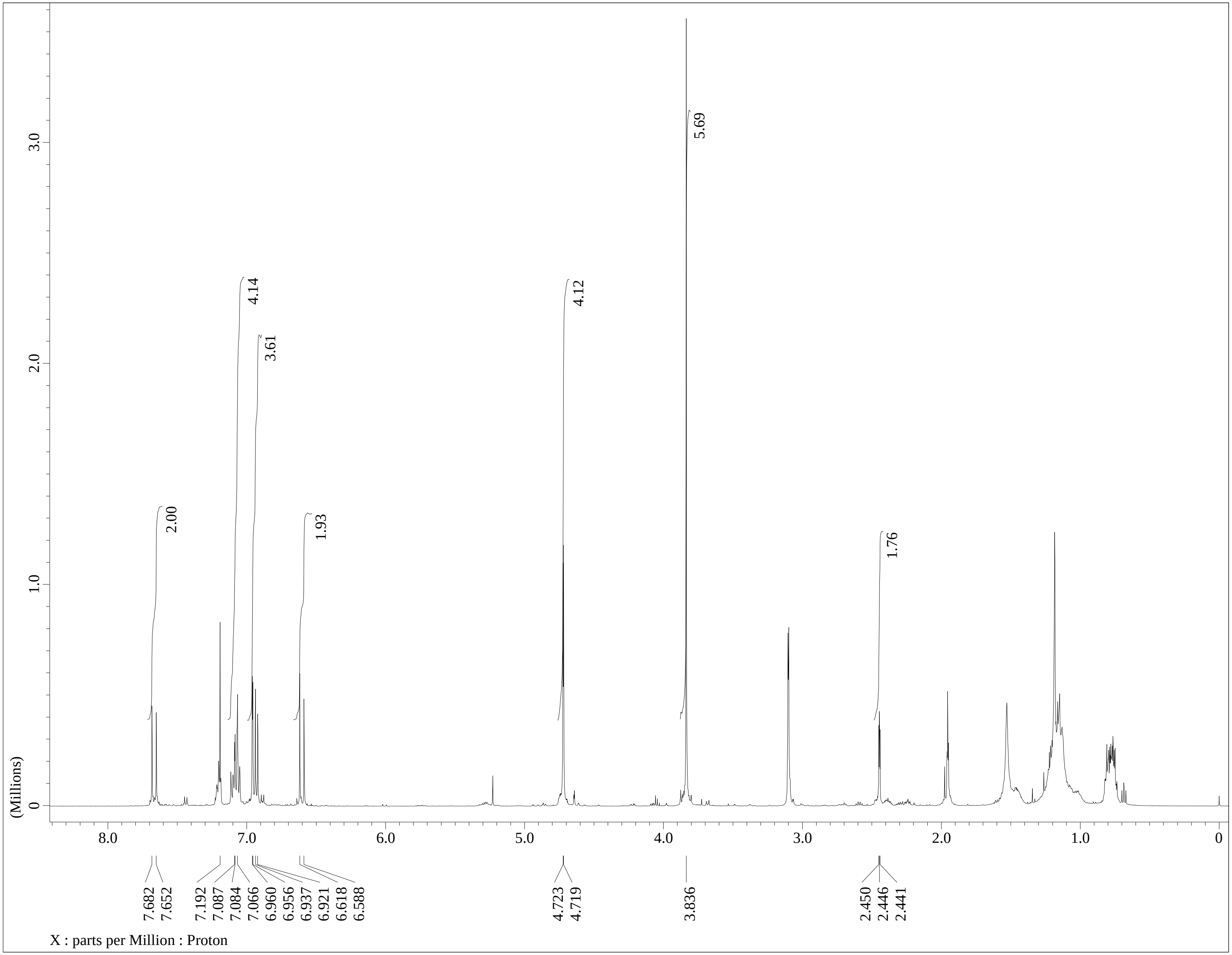

Spectra 5: ¹H NMR (500 MHz, $CDCl_3$) of compound **5**

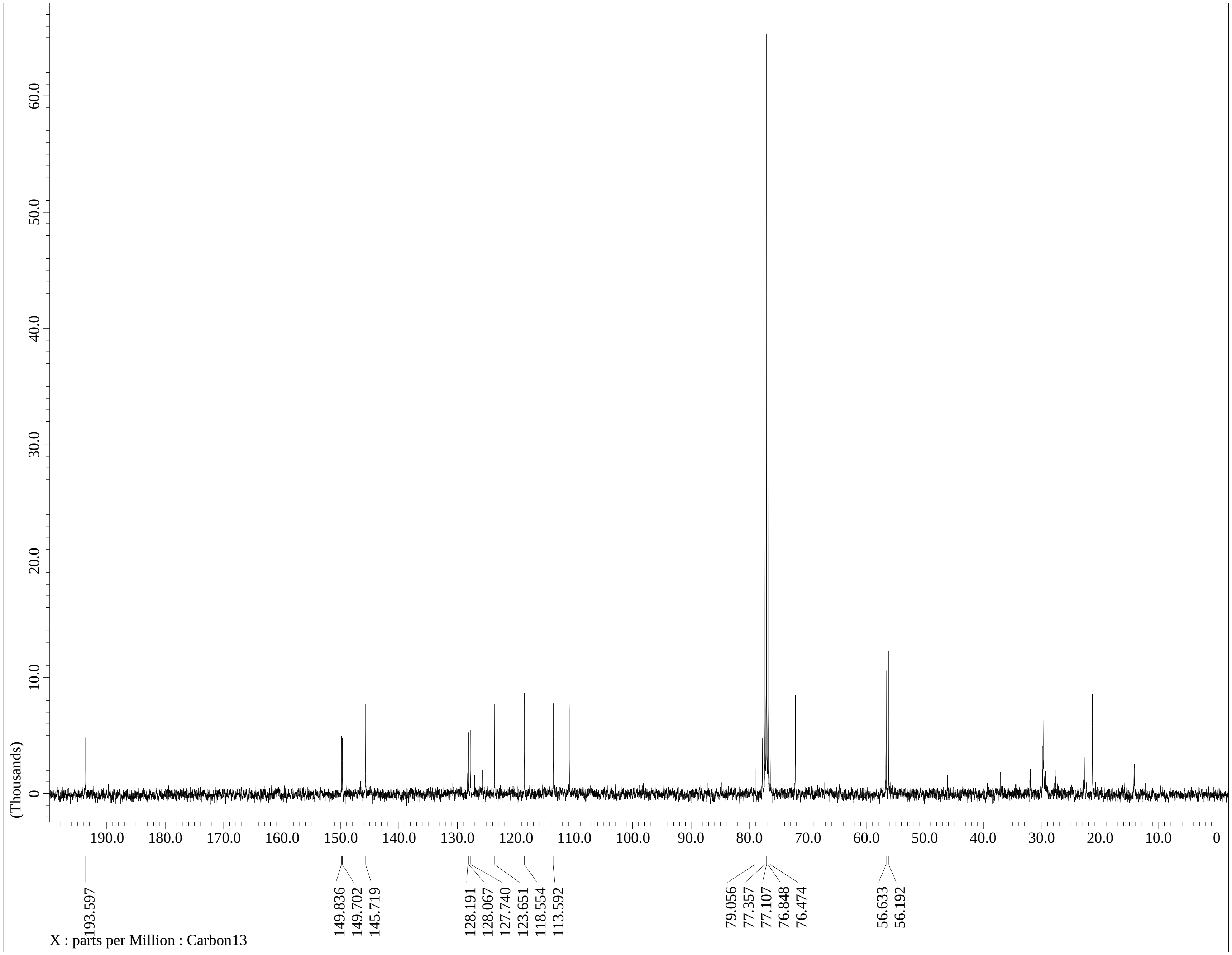

Spectra 6: ^{13}C NMR (125 MHz, $CDCl_3$) of compound **5**

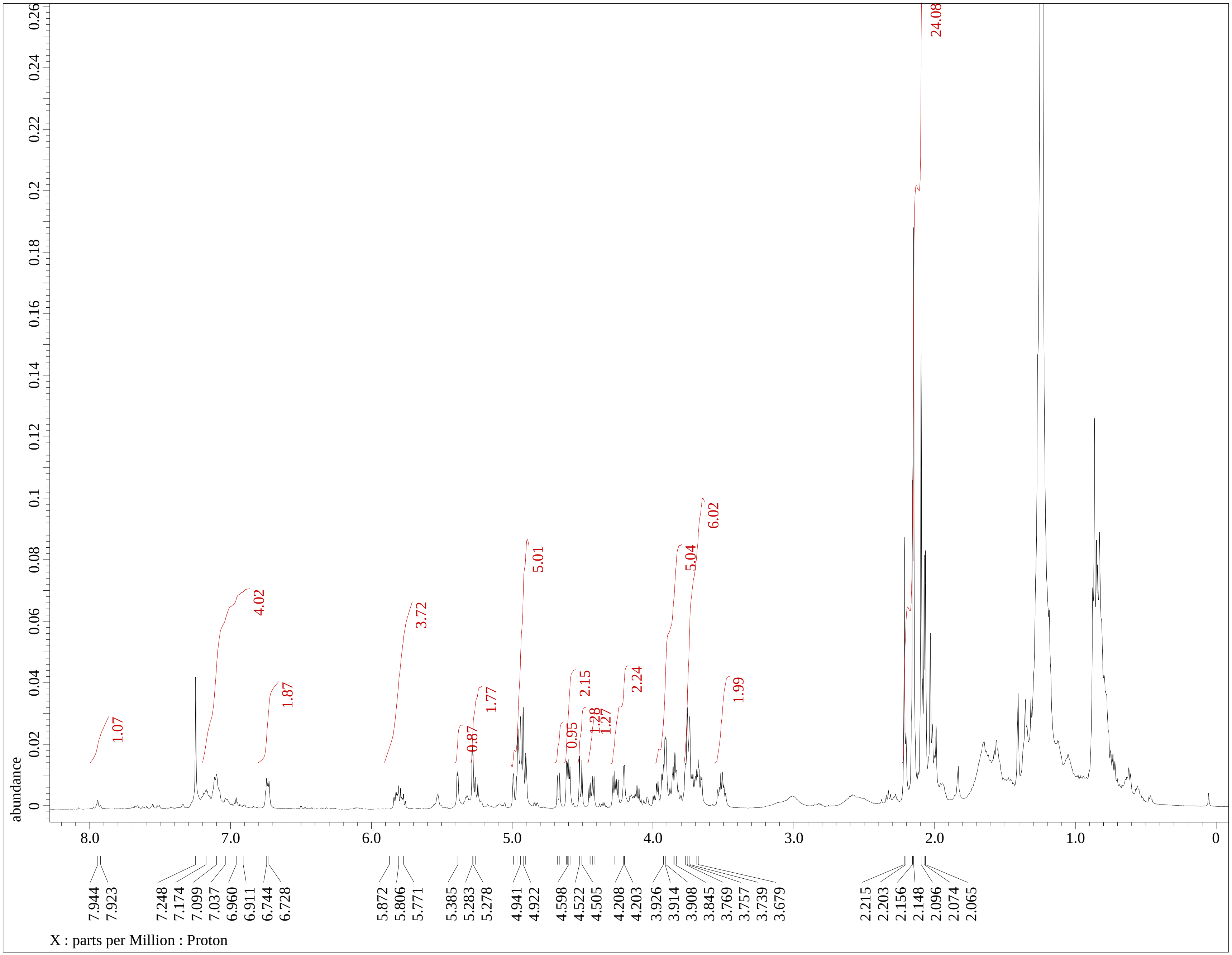

Spectra 7: ¹H NMR (500 MHz, $CDCl_3$) of compound **6**

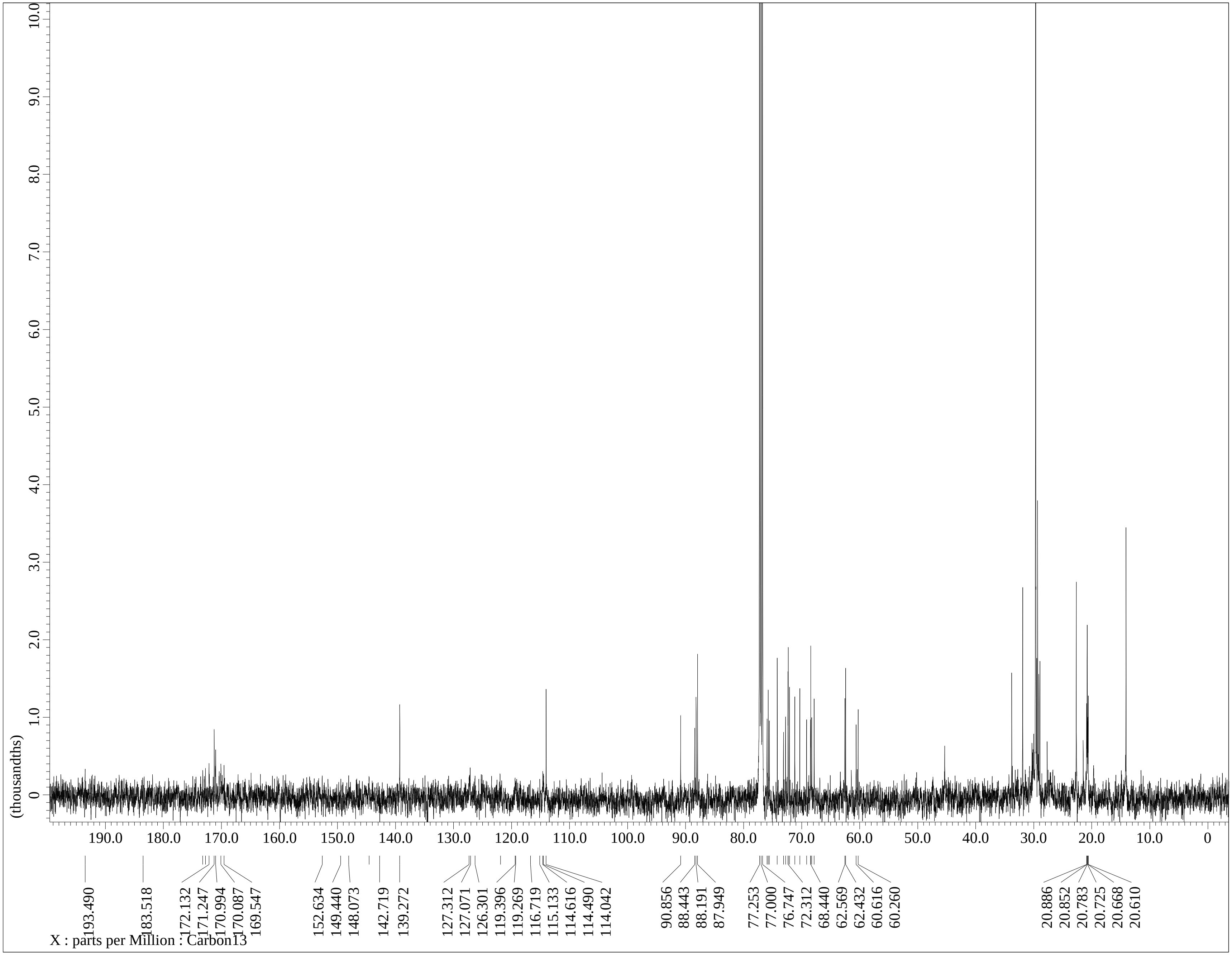

Spectra 8: ^{13}C NMR (125 MHz, $CDCl_3$) of compound **6**

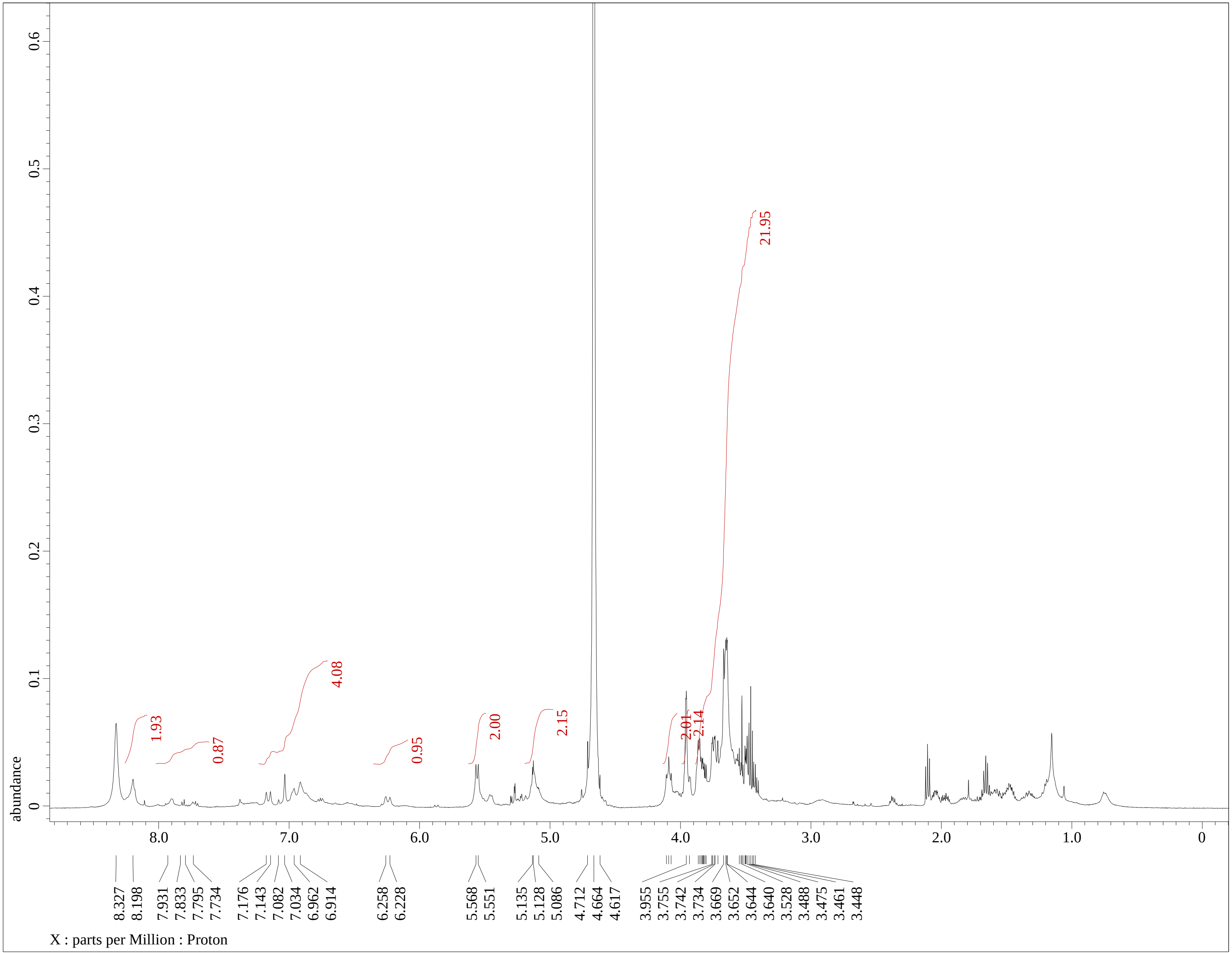

Spectra 9: Spectra 1: ^{1}H NMR (500 MHz, D_2O) of compound **7**

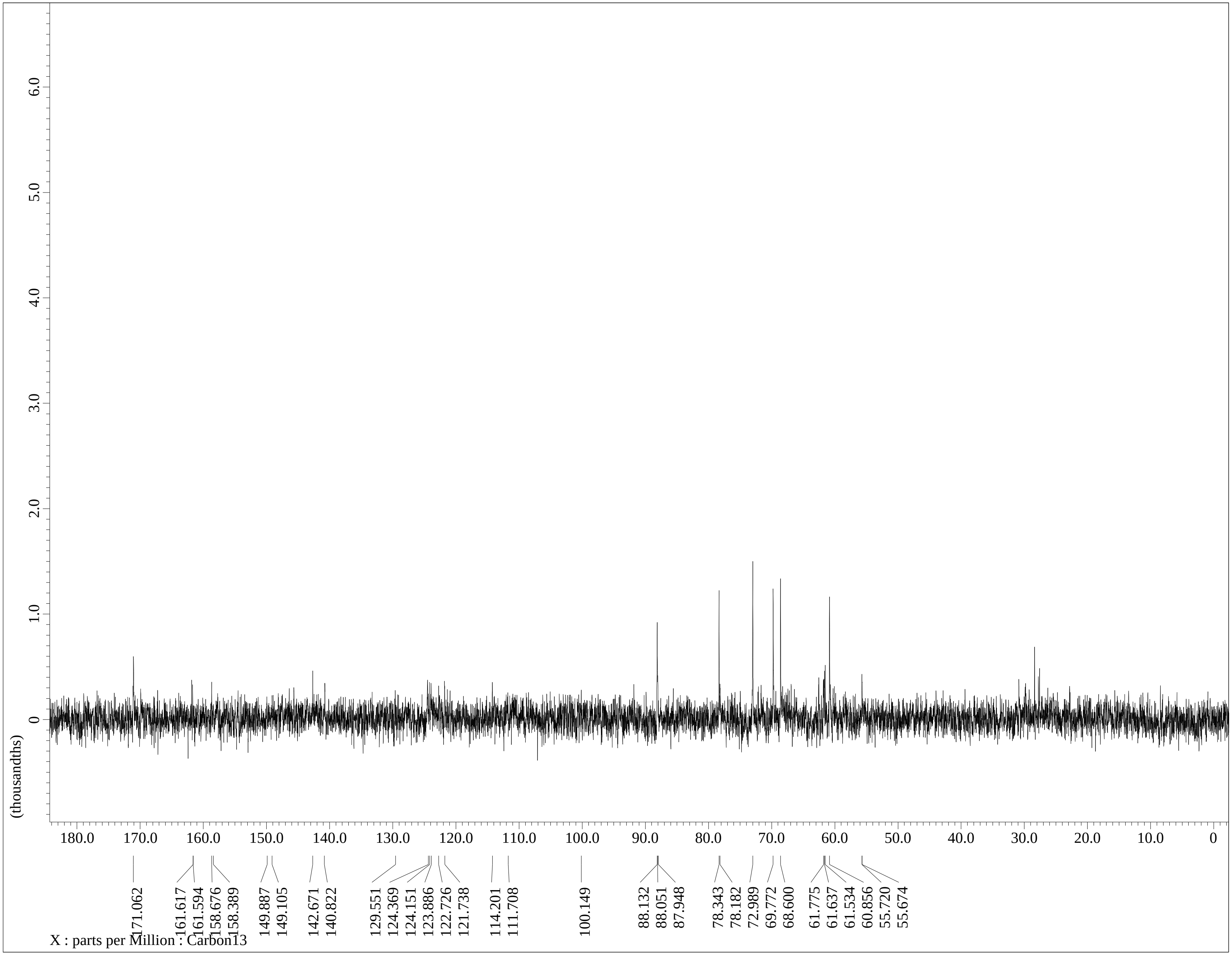

Spectra 10: ^{13}C NMR (125 MHz, D_2O) of compound **7**

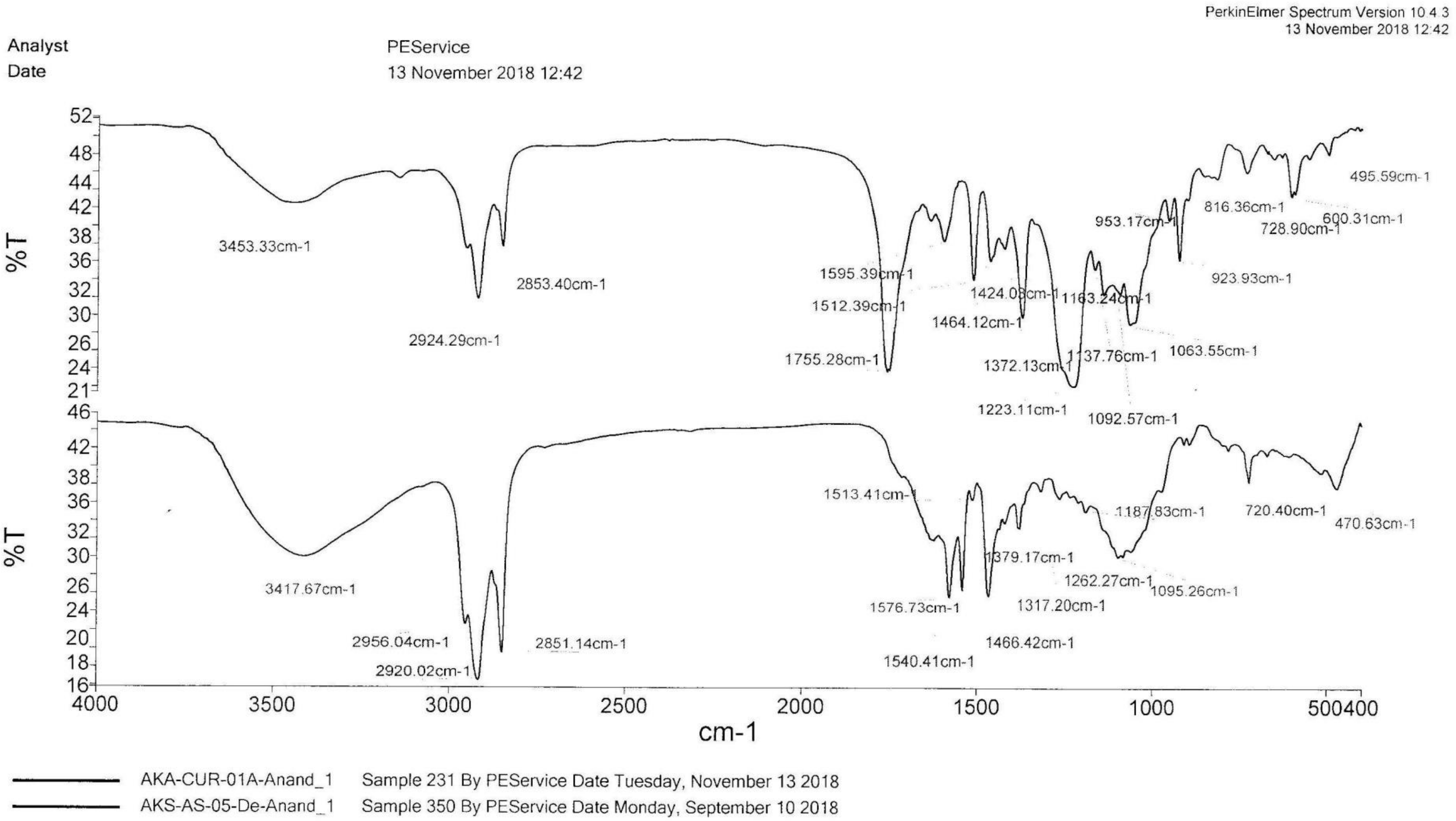

IR Spectra of compound 6 and 7

Supplementary information 3:

***In vivo* evaluation of cytotoxicity:**

Biochemical Profiling from serum and tissues:

We collected the whole blood (~1.7 ml) of the euthanized rats in EDTA blood collecting vials. Serum was separated and subjected for kit based assay of alanine aminotransferase (ALT), aspartate aminotransferase (AST), glucose, and urea using colorimetric assay kits procured from Accurex Biomedical, India. We estimated total haemoglobin using Haemocor D kit. SOD and catalase were manually assayed for serum. Haematological parameters like red blood cell counts (RBCs), white blood cells (WBCs), platelet count (PLT), mean corpuscular hemoglobin concentration (MCHC), mean corpuscular hemoglobin (MCH), mean corpuscular volume (MCV), and hematocrit (HCT) were evaluated using hematological auto analyzer (MS-9-3 France). From the liver and kidney tissues, we further assayed lipid peroxidation levels along with SOD and catalase activities.

Protein estimation

The total protein concentration was quantified by Bradford method sourcing bovine serum albumin (BSA) as standard. Briefly, BSA was dissolved in MilliQ water and diluted for the calibration curve, and 10 µl of the separated supernatant (from the tissue homogenate as well as serum) was dispensed in TCP wells. The 200 µl of the diluted Bradford reagent was added to each wells and incubated for 30 mins at room temperature. The absorbances were read at 595nm on Synergy H1 Hybrid Multi-Mode Reader. The experiment was performed in triplicate for each sample.

Lipid peroxidation assay

We assayed the malondialdehyde content, a measure of lipid peroxidation, in the form of thiobarbituric acid-reactive substances (TBARS) by the method illustrated elsewhere. Briefly, to 500 µl of supernatant equal volume of 10% trichloroacetic acid (500 µl) was added and spun at 1,000 X g for 15 min. From the thus obtained supernatant, 500 µl was harvested and added with an equal volume of 50% glacial acetic acid and 1 ml of 0.67% thiobarbituric acid and incubated for 15 mins in boiling water bath. After cooling, absorbances were read at 532 nm. Thiobarbituric acid-reactive substances were quantified using an extinction coefficient of 1.56 X 105 M^{-1} cm^{-1} and expressed as nanomole of malondialdehyde per milligram protein.

Superoxide dismutase assay:

The SOD assay was performed in accordance with the method described by Beauchamp and Fridovich. Three tubes were prepared namely sample tube(s), control tube, and blank. Reaction mixture in sample tube contained SOD buffer (pH 7.8), 130mM L-methionine (300 µl), 10 times diluted homogenate (250 µl), 750µM Nitroblue tetrazolium chloride (NBT, 150µl), 0.5mM EDTA (75µl) and 60µM riboflavin (100 µl). The control tube contained no homogenate while the blank tube was devoid of riboflavin. The tubes were kept under fluorescent light for 10 min to obtain a blue colored formazan. The obtained absorbances were read at 560nm.

Catalase assay

We assayed catalase activity by monitoring the rate of decomposition of H_2O_2 described previously. In brief, the reaction mixture (1ml) comprised of 900 µl of 50mM catalase buffer (pH 7.0) and 100 µl-diluted homogenate. The reaction was initiated by the addition of 30mM hydrogen peroxide (H_2O_2) and absorbance was measured at 240 nm for 3 min. Enzyme activity expressed in terms of micromole/min/mg protein.

Table T1. Body weight indices of control and test group rats. Data represent (mean ± S.D.) (n=6).

**Group**	**Initial body weight (gm)**	**Body weight after 48 hrs. (gm)**	**Body weight on day14 (gm)**
**Group I**	106 ± 4.47	105 ± 6.1	132.8± 2.71
**Group II**	109.3 ± 5.9	101 ± 6.2	114.6± 4.13
**Group III**	106.5 ± 5.34	106.9 ± 8.2	133.5 ± 2.7
**Group IV**	105.4 ± 4.016	106.1 ± 8.04	130.4 ± 5.12
**Group V**	104.5 ± 5.16	109 ± 6.1	134.5 ± 3.94
**Group VI**	107.3 ± 4.25	109.6 ± 4.2	128.8 ± 7.36

Table T2: Vital organ indices and Organ/body weight ratio of different groups on 15th day. Data represent (Mean ± S.D.) (n=6).

Groups	Liver (gm)	Liver/ Body weight ratio	Kidney (left) (gm)	Kidney/ Body weight ratio	Spleen (gm)	Spleen/ Body weight ratio
**Group I**	5.19 ± 0.16	0.03908 ± 0.002	0.45 ± 0.027	0.00388 ± 0.0003	0.44 ± 0.014	0.00331 ± 0.00012
**Group II**	3.51 ± 0.42	0.03062 ± 0.0019	0.30 ± 0.014	0.00262 ± 0.0001	0.30 ± 0.014	0.00261 ± 0.00016
**Group III**	5.23 ± 0.23	0.03917 ± 0.001	0.46 ± 0.025	0.00345 ± 0.0002	0.42 ± 0.017	0.00314 ± 0.00012
**Group IV**	5.07 ± 0.38	0.03888 ± 0.002	0.43 ± 0.047	0.00329 ± 0.0004	0.40 ± 0.035	0.00306 ± 0.00017
**Group V**	5.41 ± 0.22	0.04022 ± 0.0008	0.45 ± 0.021	0.00345 ± 0.0002	0.41 ± 0.018	0.00304 ± 0.00014
**Group VI**	5.31 ± 0.27	0.04122 ± 0.0018	0.41 ± 0.067	0.00318 ± 0.0002	0.42 ± 0.039	0.00326 ± 0.00036

Table T3: Kit based serological profiling of control and test group rats. Data represent (Mean ± S.D.) (n=6).

Parameters	Group I	Group II	Group III	Group IV	Group V	Group VI
**Glucose (mg/ml)**	102.3 ± 11.2	105.5 ± 6.4	102.7 ± 9.3	103.1 ± 10.1	102.5 ± 9.6	105.1 ± 9.7
**GPT (U/L)**	108.2 ± 6.7	141.6± 5.45	110.3 ± 5.1	108.9 ± 6.2	111.3 ± 6.4	113.2 ± 4.2
**GOT (U/L)**	72.4 ± 5.2	90.61 ± 3.6	69.7 ± 6.56	70.2 ± 3.57	69.4 ± 8.1	70 ± 4.5
**SOD (U/mg protein)**	2.17 ± 0.27	1.25 ±0.12	2.11 ± 0.13	2.09 ± 0.14	2.11 ± 0.11	2.07 ± 0.12
**Catalase (U/mg protein)**	20.45 ±1.61	14.84 ± 2.2	20.5 ± 1.43	19.65 ±1.87	21.3 ± 2.14	20.89 ± 2.06
**Hemoglobin(g/dl)**	12.13 ±0.45	11.56 ± 0.24	12.05 ± 0.31	12.14 ± 0.82	12.21 ± 0.43	12.17 ± 0.24
**Urea (mg/dl)**	46.5 ± 6.9	62.6 ± 4.8	44.21 ± 4.4	44.09 ± 7.6	45.7 ± 6.2	44.89 ± 8.2
**Creatinine (mg/dl)**	0.38 ± 0.022	0.69 ± 0.013	0.406 ± 0.21	0.39 ± 0.24	0.401 ± 0.32	0.405 ± 0.27

Table T4: Protein estimation from liver, kidney, and serum among different groups of rats. Each experiment was done in triplicate. Data represents Mean±SD; n=6

Protein (mg/ml)	Group I	Group II	Group III	Group IV	Group V	Group VI
	Protein (mg/ml)	Protein (mg/ml)	Protein (mg/ml)	Protein (mg/ml)	Protein (mg/ml)	Protein (mg/ml)
**Liver**	4.876± 0.0592	4.436±0.213	4.431±0.0621	4.632±0.0947	4.584±0.248	4.701±0.106
**Kidney**	7.614±0.0341	7.251±0.0681	7.515±0.0942	7.647±0.0355	7.428±0.0573	7.515±0.0474
**Serum**	6.059±0.2613	4.751±0.2423	5.875±0.2426	5.758±0.3171	6.085±0.2374	5.985±0.3268

Table T5: Activities of enzymes in liver and kidney tissues respectively (A) lipid peroxidation as the level of TBARS/ MDA concentration (B) activity of superoxide dismutase (C) activity of catalase in various groups of rats. Each experiment was done in triplicate. Data represents Mean±SD; n=6

	**Parameters**					
**Groups**	**Lipid Peroxidation***		**Superoxide dismutase$**		**Catalase#**	
	**Liver**	**Kidney**	**Liver**	**Kidney**	**Liver**	**Kidney**
**Group I**	0.7465±0.0322	0.4891±0.0142	3.145±0.1652	2.084±0.0534	332.8±4.243	144.6±3.417
**Group II**	0.8945±0.0418	0.5618±0.0254	2.251±0.1564	1.572±0.0724	292.8±8.451	98.85±6.394
**Group III**	0.7461±0.0257	0.4904±0.0216	3.064±0.1443	1.964±0.05091	333.5±7.304	138.5±5.412
**Group IV**	0.7384±0.0258	0.4837±0.0242	2.987±0.1257	2.041±0.0261	331.6±9.413	137.81±4.823
**Group V**	0.7541±0.0354	0.4964±0.0294	3.084±0.1261	2.054±0.0384	334.1±5.602	140.3±2.107
**Group VI**	0.7571±0.0327	0.4951±0.0157	3.137±0.1413	2.069±0.0153	333.7±6.176	142.5±5.614

*nM/mg protein, $µmole/min/mg protein, #µmole/min/mg protein

Table T6: Automated hematological profiling of control and test group rats. Data represent (Mean ± S.D.) (n=6).

Groups	Hb (gm dl^{-1})	RBC (10^3 UL^{-1})	WBC (10^3 UL^{-1})	PLT (10^3 UL^{-1})	HCT (%)	MCHC (%)	MCH (pgm)	MCV (FI)
**Group-I**	11.87 ± 0.64	8.29 ± 0.47	4.41 ± 0.45	5.22 ± 0.14	34.83 ± 1.34	34.3 ± 0.41	15.68 ± 0.32	46.3 ± 1.08
**Group-II**	10.97 ± 0.41	6.56 ± 0.68	3.57 ± 0.18	4.03 ± 0.82	31.35 ± 1.43	30.94 ± 0.52	12.51 ± 0.17	42.54 ± 1.31
**Group-III**	12.48 ± 0.73	8.21 ± 0.49	4.39 ± 0.14	5.34 ± 0.26	35.36 ± 0.14	33.84 ± 0.35	15.12 ± 0.43	46.14 ± 0.47
**Group-IV**	12.36 ± 0.67	8.22 ± 0.12	4.36 ± 0.24	5.52 ± 0.21	35.89 ± 1.03	34.17 ± 0.58	15.39 ± 0.12	46.29 ± 0.48
**Group-V**	11.89 ± 0.34	8.47 ± 0.13	4.35 ± 0.43	5.17 ± 0.34	36.12 ± 0.61	34.69 ± 0.23	15.57 ± 0.42	46.07 ± 0.56
**Group-VI**	11.88 ± 0.94	8.34 ± 0.24	4.62 ± 0.25	5.25 ± 0.27	35.94 ± 0.15	34.81 ± 0.14	15.65 ± 0.67	46.44 ± 0.33

Table T7: Modified Minimum inhibitory concentration of Vancomycin, Meropenem, and Ciprofloxacin upon administration with native curcumin (8 µg/ml) against MDR pathogens

**Isolates**	**Vancomycin**<br>**Modified MIC (µg/ml)**	**Meropenem**<br>**Modified MIC (µg/ml)**	**Ciprofloxacin**<br>**Modified MIC (µg/ml)**
Staphylococcus aureus **(MRSA, 2862/2019)**	1	-	32
Klebsiella pneumoniae **(10825/2019)**	512	128	64
Escherichia coli **(507/2019)**	512	64	32
Pseudomonas aeruginosa **(2412/2019)**	1024	128	64

Supplementary information 4:

Comaparative evaluation of curcumin and vancomycin combination on *Klebsiella pneumoniae* viability at different concentrations

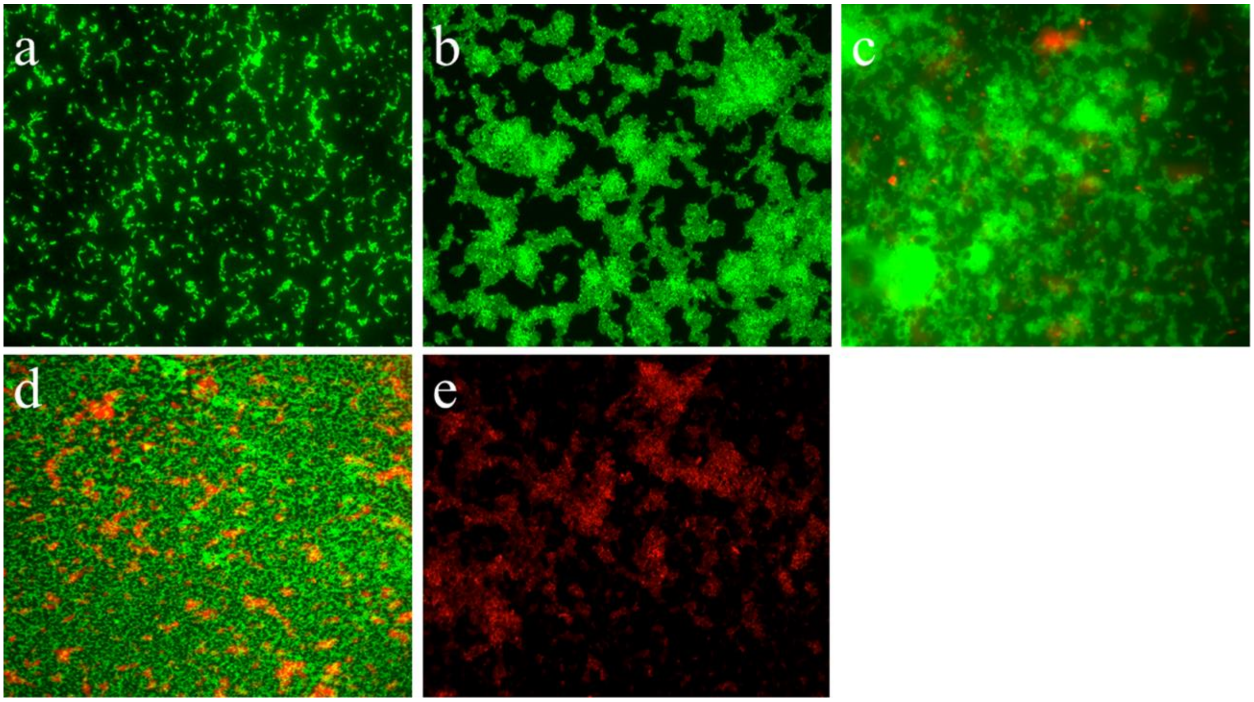

S4: Confocal Microscopic validation of synergism between soluble curcumin and vancomycin over multidrug resistant clinical *Klebsiella pneumoniae***.** (a) Treated *Klebsiella pneumoniae* at t=0 hrs with native curcumin (128 µg/ml) (b) Treated *Klebsiella pneumoniae* at t=18 hrs. (c) Native curcumin (128 µg/ml) and vancomycin (2 µg/ml) treated *Klebsiella pneumoniae* at t=18 hrs. (d) Native curcumin (128 µg/ml) and vancomycin (4 µg/ml) treated *Klebsiella pneumoniae* at t=18 hrs. (e) Positive control i.e. Meropenem (4 µg/ml) treated *Klebsiella pneumoniae* at t=18 hrs. (All confocal images were analyzed and processed using ZenBlue imaging software).

Supplementary information 5:

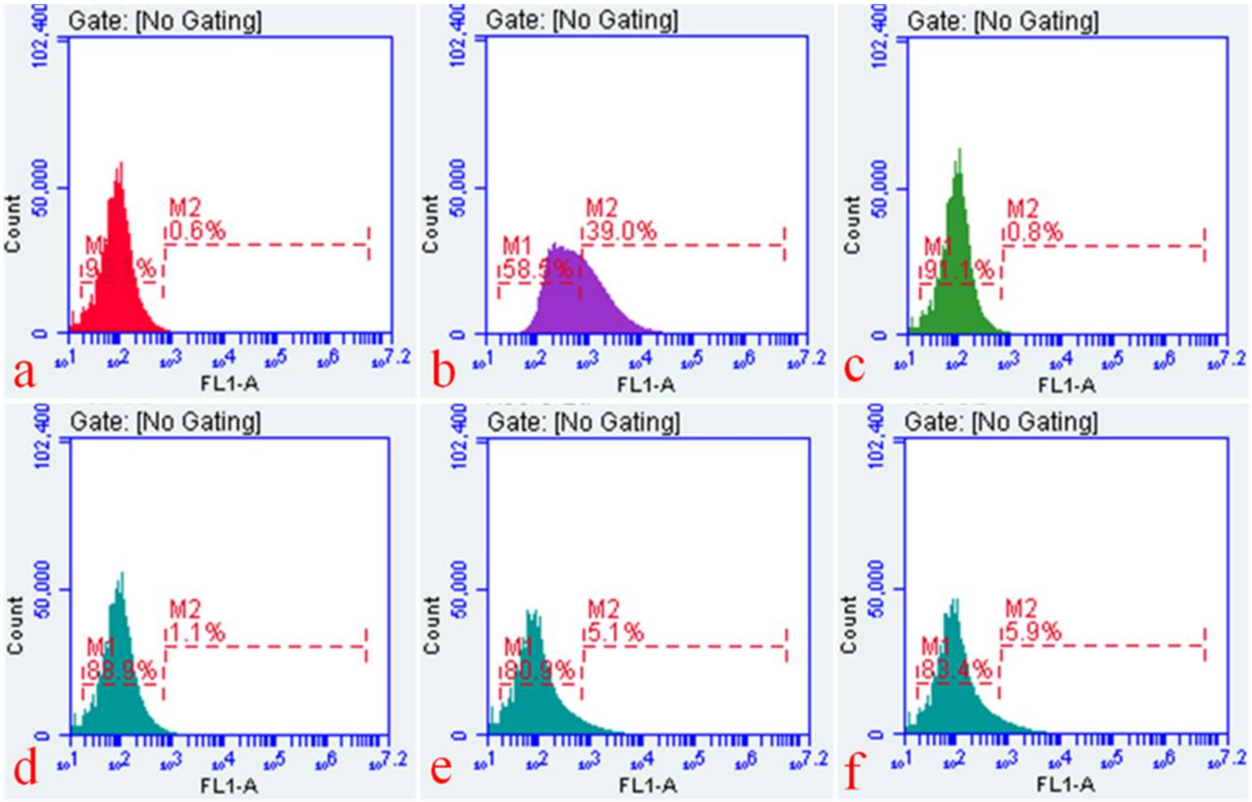

Flow cytometry analyses of *Klebsiella pneumoniae* with DPH to explore the changes in its membrane dynamics in the groups treated with discrete sets of compounds and their combination. The symbols, pattern and colours used here remained the same for every panel. Total 10, 00,000 cells were taken into account for each analysis. (a) Histograms of untreated logarithmic *Klebsiella* cells labeled with DPH. (b) Histograms of native curcumin (256 µg/ml) treated *Klebsiella* isolates labeled with DPH. There is significant right shifting of the population indicating substantial acyl shifting/ change in membrane dynamics. (c) Histograms of vancomycin (256 µg/ml) treated *Klebsiella* isolates labeled with DPH. There is minor increase in the intensity compared to the untreated and mild right shifting of the population, indicating the negligible acyl shifting, or the change in membrane dynamics. (d) Histograms of native curcumin and vancomycin combination (8 µg/ml and 32 µg/ml) of the treated *Klebsiella* isolates labeled with DPH. There is minor increase in the intensity compared to the untreated and mild right shifting of the population (1.1%), indicating the negligible acyl shifting, or the change in membrane dynamics. (e) Histograms of native curcumin and vancomycin combination (8 µg/ml and 64 µg/ml) treated *Klebsiella* isolates labeled with DPH. The panels show the minor right shifting indicating increase in cell population (5.1%) that had taken up DPH designating increased alteration in membrane lipids. (f) Histograms of native curcumin and vancomycin combination (8 µg/ml and 128 µg/ml) treated *Klebsiella* isolates labeled with DPH. The panels show the minor right shifting indicating increase in cell population (5.9%) that had taken up DPH designating relatively increased alteration in membrane lipids. (Histograms were generated and analyzed using BD Accuri C6 Software).

Supplementary information 6:

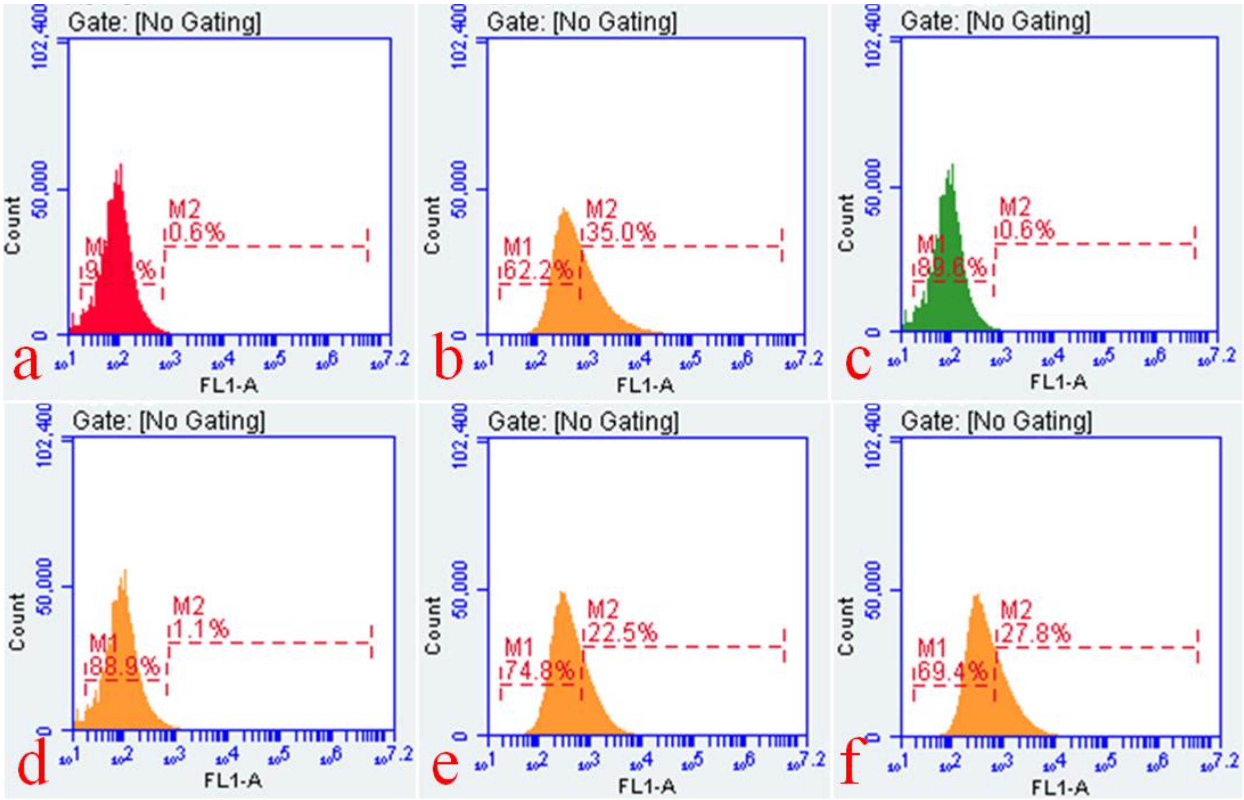

Flow cytometry analysis of *Klebsiella pneumoniae* with membrane potential-sensitive dye, $DiSC_3$-5 to explore the alterations in its membrane potential. The symbols, pattern and colours used here remained the same for every panel. Total 10, 00,000 cells were taken into account for each analysis. (a) Histograms of untreated logarithmic *Klebsiella* cells labeled with $DiSC_3$-5. (b) Histograms of native curcumin (256 µg/ml) treated *Klebsiella* isolates labeled with $DiSC_3$-5. There is significant right shifting (35%) of the population indicating substantial acyl shifting/ change in membrane dynamics. (c) Histograms of vancomycin (256 µg/ml) treated *Klebsiella* isolates labeled with $DiSC_3$-5. There is minor increase (0.6%) in the intensity compared to the untreated and mild right shifting of the population, indicating the negligible acyl shifting, or the change in membrane dynamics. (d) Histograms of native curcumin and vancomycin combination (8 µg/ml and 32 µg/ml) of the treated *Klebsiella* isolates labeled with $DiSC_3$-5. There is minor increase in the intensity compared to the untreated and mild right shifting of the population (1.1%), indicating the negligible change in membrane potential. (e) Histograms of native curcumin and vancomycin combination (8 µg/ml and 64 µg/ml) treated *Klebsiella* isolates labeled with $DiSC_3$-5. The panels show the minor right shifting indicating increase in cell population (22.5%) that had taken up $DiSC_3$-5 designating increased alteration in membrane potential. (f) Histograms of native curcumin and vancomycin combination (8 µg/ml and 128 µg/ml) treated *Klebsiella* isolates labeled with $DiSC_3$-5. The panels show the minor right shifting indicating increase in cell population (27.8%) that had taken up $DiSC_3$-5 designating relatively increased alteration in membrane potential. (Histograms were generated and analyzed using BD Accuri C6 Software)

Supplementary information 7:

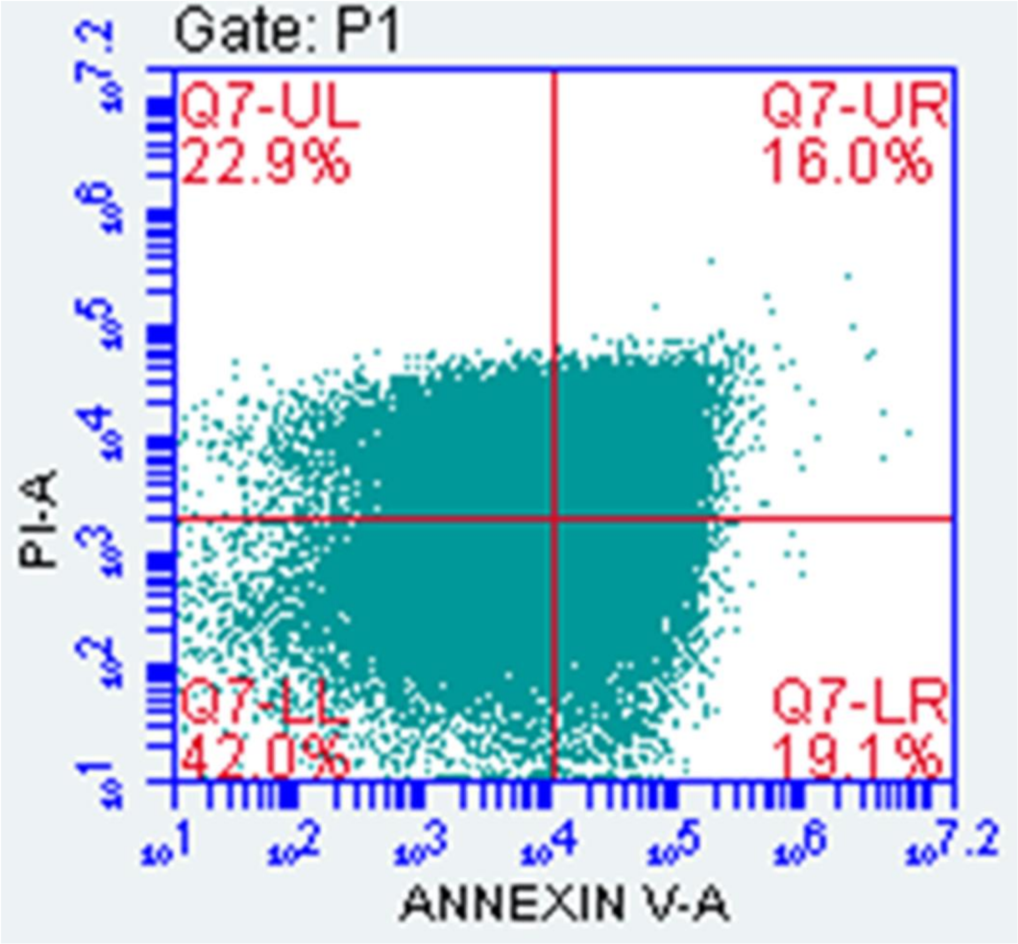

Flow cytometry based evaluation of native curcumin-vancomycin (8 µg/ml + 32 µg/ml) mediated apoptosis-like phenomena utilizing Annexin-V and PI dual staining. Only 16% of the total population is shifted to the third quadrant indicating poor effectivity of the combination in triggering apoptosis in the cell population. (Histogram was generated and analyzed using BD Accuri C6 Software)
